# Reviewing Challenges of Predicting Protein Melting Temperature Change Upon Mutation Through the Full Analysis of a Highly Detailed Dataset with High-Resolution Structures

**DOI:** 10.1007/s12033-021-00349-0

**Published:** 2021-06-08

**Authors:** Benjamin B. V. Louis, Luciano A. Abriata

**Affiliations:** 1 Master of Life Sciences Engineering, School of Life Sciences, École Polytechnique Fédérale de Lausanne, CH-1015 Lausanne, Switzerland; 2 Laboratory for Biomolecular Modeling, School of Life Sciences, École Polytechnique Fédérale de Lausanne, and Swiss Institute of Bioinformatics, CH-1015 Lausanne, Switzerland; 3 Protein Production and Structure Core Facility, School of Life Sciences, École Polytechnique Fédérale de Lausanne, CH-1015 Lausanne, Switzerland

**Keywords:** Protein engineering, Protein design, Mutagenesis, Machine learning, Protein stability, Mutation

## Abstract

Predicting the effects of mutations on protein stability is a key problem in fundamental and applied biology, still unsolved even for the relatively simple case of small, soluble, globular, monomeric, two-state-folder proteins. Many articles discuss the limitations of prediction methods and of the datasets used to train them, which result in low reliability for actual applications despite globally capturing trends. Here, we review these and other issues by analyzing one of the most detailed, carefully curated datasets of melting temperature change (ΔTm) upon mutation for proteins with high-resolution structures. After examining the composition of this dataset to discuss imbalances and biases, we inspect several of its entries assisted by an online app for data navigation and structure display and aided by a neural network that predicts ΔTm with accuracy close to that of programs available to this end. We pose that the ΔTm predictions of our network, and also likely those of other programs, account only for a baseline-like general effect of each type of amino acid substitution which then requires substantial corrections to reproduce the actual stability changes. The corrections are very different for each specific case and arise from fine structural details which are not well represented in the dataset and which, despite appearing reasonable upon visual inspection of the structures, are hard to encode and parametrize. Based on these observations, additional analyses, and a review of recent literature, we propose recommendations for developers of stability prediction methods and for efforts aimed at improving the datasets used for training. We leave our interactive interface for analysis available online at http://lucianoabriata.altervista.org/papersdata/proteinstability2021/s1626navigation.html so that users can further explore the dataset and baseline predictions, possibly serving as a tool useful in the context of structural biology and protein biotechnology research and as material for education in protein biophysics.

## Introduction

Quantitative prediction of the effects of mutations on protein stability is a yet unsolved problem of key relevance in structural biology, molecular evolution, and protein biotechnology [1–8] and part of the larger problem of predicting the phenotypic effects of genomic variation [2, 9, 10]. Developing such predictive models requires sufficiently large training datasets describing the quantitative effects of mutations on protein stability. Although it is not clear how large is enough, datasets likely need to properly represent all possible amino acid substitutions and cover a vast range of structural scenarios. Many groups have, thus, compiled over the years datasets of experimentally determined stability changes upon mutation, where the effect is quantified mainly as the change in thermodynamic stability, i.e., in (un)folding free energy (ΔΔGu) or as the change in melting temperature (ΔTm). These datasets typically contain full records for only one of either ΔΔGu or ΔTm, which is not a minor issue because ΔΔGu and ΔTm are not necessarily correlated and, thus, cannot always be exchanged for modeling purposes [11]. And to date, datasets are dominated by ΔΔGu data rather than the easier-to-interpret ΔTm, pragmatically more useful in structural biology and protein biotechnology.

The most important datasets of protein stability effects upon mutation, combining both ΔTm and ΔΔGu data, have been derived by curation and literature-based completion of the ProTherm [12] and ThermoMut [13] databases, which are quite extensive but also heterogeneous and incomplete in many records relevant to the problem of predicting Tm changes upon mutation. For example, for certain proteins only, the wild-type versions have stability data available; in other cases, the parameters for wild type and mutants were obtained in quite different conditions. Besides, some records lack connections to structures, thus, being useful only for sequence-based predictions, naturally less accurate than structure-based estimations. A very recent review by Mazurenko [14] pinpoints other problems in ProTherm,^1^ discussing also the most important datasets available as of 2020 and putting forward a new dataset. Another excellent review by Sanavia et al. also discusses ProTherm-derived datasets as well as several popular predictors of mutational effects on protein stability [1].

A recurrent problem in the field of mutant stability prediction is that even though every new program or server claims superiority over others reporting good correlation coefficients and low mean-square errors between predicted and known ΔΔGu or ΔTm, subsequent tests by third groups always reveal poorer performances. The latest such evaluations [1, 15–18] conclude that (i) training datasets are too small and unbalanced, biased towards destabilizing mutations and not smoothly covering all possible amino acid replacements, (ii) they are quite redundant and dominated by few protein families, thus, possibly biased to certain types of proteins, (iii) the stability parameters are available in quite diverse conditions, and (iv) many models seem to be overfit, introducing biases on the predictions to the extreme that they even fail to predict those mutations that are reversed relative to what is available in the training dataset (i.e., backwards mutant-to-wild-type predictions). These works show that, on large testing datasets, most methods do show some correlations between experimental and predicted values, and that the distribution of predicted stability changes does follow the shape of natural distributions, thus, turning out useful for large-scale analyses where only trends are relevant, as in coarse modeling of protein evolution [19]. However, the works also show that specific predictions or even predictions for large datasets of mutations on proteins under-represented in the training dataset are still too off for practical applications. Just to mention two recent cases, the reader is referred to the works on guanylate Kinase by McGuinness et al. [17] and β-glucosidase by Huang et al.[20].^2^ Notably, a detailed study on haloalkane dehalogenase by Beerens et al. [21] showed that even in the few cases where mutations designed to stabilize a protein were successful, they largely optimized enthalpy but not entropic contributions, which are as important tuners of protein stability in natural protein variants, thus, pointing to yet another shortcoming of the methods for stability prediction. Another important problem is that given the larger number of destabilizing over stabilizing mutations in all datasets, most methods are biased to destabilization and, thus, do not reproduce the expected symmetry for forwards and backward mutations. Recently, interesting ways to treat this problem on ΔΔGu predictions, possibly adaptable to ΔTm predictions, have been proposed [16, 22, 23].

While oligomerization, membrane integration or association, disordered regions, and other features naturally complicate protein stability predictions, the works mentioned above show that the problem of stability change prediction is still far from solved even for small, soluble, monomeric, well-folded proteins. Beyond the pitfalls in the training datasets and in the methods themselves as summarized above and discussed in many works, in this review, we focus mainly on the structural subtleties that lead to special situations of strong stabilization and destabilization as judged by ΔTm and based on high-resolution structures. We pose that these structural subtleties likely confuse the otherwise clear trends that can be modeled with simple physicochemical descriptors of the amino acid substitutions which is what most methods and programs do. Analysis of such situations in turn highlights very important aspects of having complete datasets well-balanced over all possible amino acid substitutions and structural situations. These analyses shall be of special interest to method developers, to protein designers, and structural biologists in general, to efforts aimed at improving datasets for training stability predictors, and possibly also for educational purposes.

We base our review on one of the most complete, carefully curated datasets of Tm change upon mutation, published by Pucci, Bourgeas, and Rooman in 2016 [11]. Although more datasets became available afterwards, this is, in our view, the most complete and carefully curated dataset containing high-structural information and full ΔTm data to date, with other advantages as discussed below. We first dissect the dataset in the context of other works and reviews to highlight its value and identify its limitations especially its coverage and imbalances. We then navigate the dataset through an online web app that resolves instances of each of the 20 × 19 possible substitutions against structural parameters aided by structure views and predictions of a neural network that performs similar to published methods for ΔTm prediction, serving as a baseline for the identification of interesting cases that deviate strongly from the general prediction. We, thus, identify and discuss several such cases in structural detail, building up the idea of global vs. structure-specific contributions to stability changes upon mutation and developing the sense that the general effects caused by each kind of mutation are relatively easy to capture but the effects of case-specific local structural features that induce strong (de)stabilization are not, and not even well represented in the dataset. Knowing the Tm changes induced by mutations in these examples, we can qualitatively explain them quite well in terms of protein structure considerations; however, they are very hard to be effectively predicted a priori*,* probably even harder for automated methods. Along the way and especially by the end of the review, we also elaborate on possible routes for future improvements of methods for stability prediction upon mutation and, of key relevance, of the datasets used to train them.

## The “S1626” Dataset of Small, Soluble, Monomeric, Globular Proteins by Pucci et al.

**Fig. 1:**
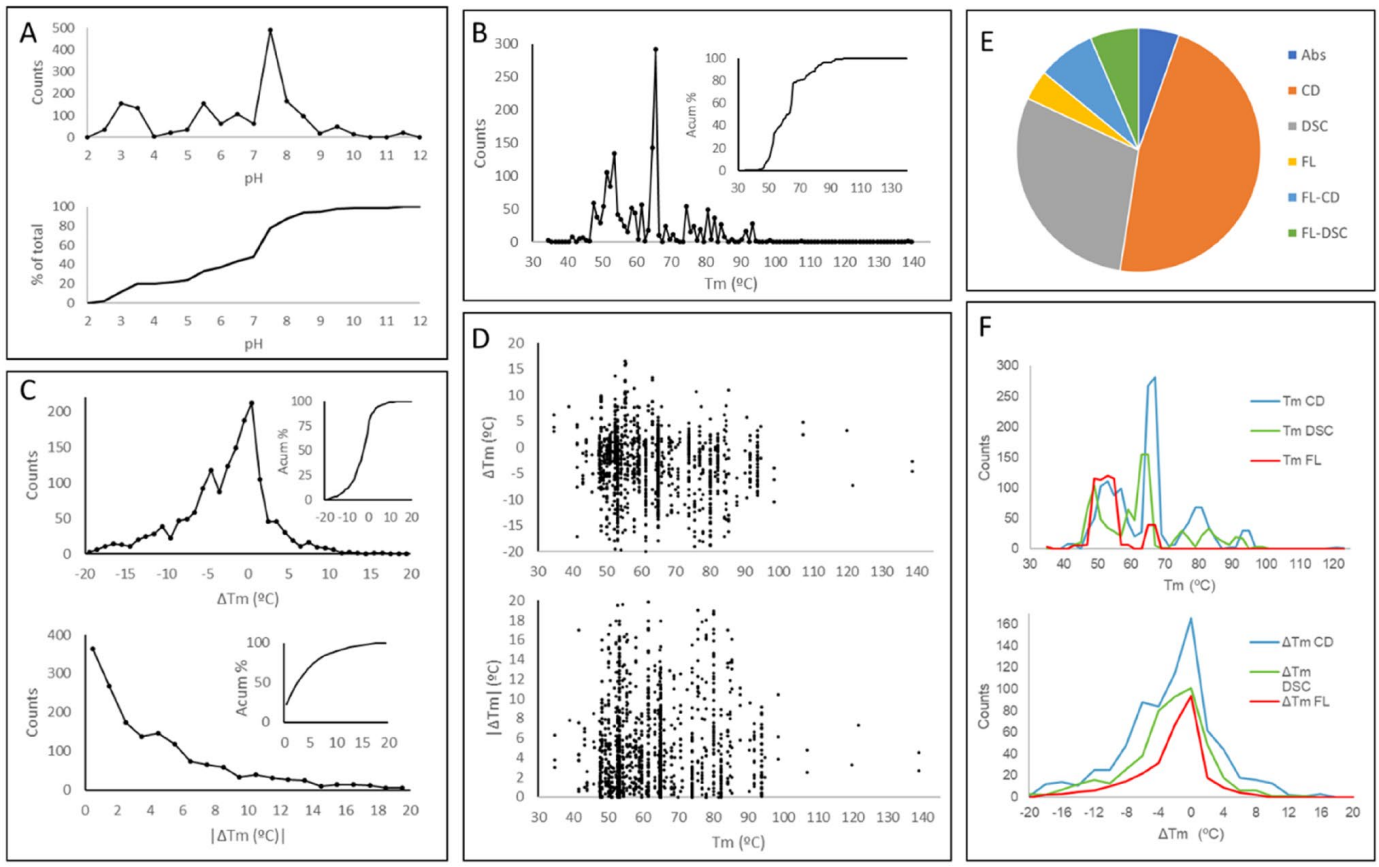
Description of the S1626 dataset of ∆Tm upon mutation compiled by Pucci et al. (**A**) Distribution of pH conditions at which ∆Tm were measured. (**B**) Distribution of reference Tm values, i.e., before mutation (“wild type”). (**C**) Distribution of ∆Tm and |∆Tm| values. (**D**) ∆Tm and |∆Tm| resolved against reference Tm. (**E**) Representation of each technique used to track protein unfolding (Abs = absorption, CD = circular dichroism, DSC = differential scanning calorimetry, FL = fluorescence). (**F**) Distribution of Tm and ∆Tm values measured with each technique

The dataset by Pucci et al. contains full ΔTm data for 1626 mutations (hence “S1626”) from experimental measurements in 90 globular proteins of structure known at high resolution, all compact, globular, monomeric in solution at least in their wild-type forms, and known two-state folders. This, as opposed to other larger datasets that include oligomeric and transmembrane proteins, or that lack high-resolution structures or even lack structures at all, or contain only ΔΔGu data, etc., which might be useful too but do not provide a sufficiently clear basis for our analyses. Each entry of S1626 contains Tm and ∆Tm relative to wild type, flagged with the experimental techniques and conditions used to measure them, and in many cases also extended parameters that further describe the impact of the mutations on protein stability such as changes in folding free energy, enthalpy, entropy, and heat capacity. Stability measurements as close to neutral pH as possible were chosen upon construction by Pucci et al. when multiple options were available; in practice, 50% of the entries are at pH 6–8, 13% above pH 8, and 37% below pH 6 including some 20% of the total at pH 3.5 or lower (Fig. 1A). Mesostable and thermostable proteins were included, the latter showing a somewhat larger fraction of destabilizing mutations according to the authors of the dataset. On analyzing this through the distribution of Tm values (Fig. 1B), there is a group in the range from 40 to 64 °C that accounts for ~ 50% of the mutation entries. The other 50% spans the contiguous range up to 100 °C but displays very large numbers of cases around 64-65 °C, clear as a peak in the distribution in the plot. This peak arises from 182 entries for PDB 1L63 (Tm = 65.1 °C), 104 for PDB 2LZM (Tm = 65.1 °C), and 129 for PDB 1LZ1 (Tm = 64.9 °C). These 3 structures are of T4 lysozyme and together account for around 25% of the dataset. The first two are actually the same lysozyme, from the T4 bacteriophage, differing by only 2 non-synonymous mutations and superimposing within 0.16 Å all-atom RMSD, accounting for 18% of the dataset. Meanwhile, PDB 1LZ1 corresponds to human lysozyme which has a very different sequence and could, thus, be regarded as a genuinely different system. Overall, lysozyme accounts for almost 30% of the dataset, followed by ribonuclease at almost 13% and *Staphylococcus* nuclease at 8%. This dominance of one particular protein type and Tm values pose a potential bias, present in most databases that compromises the quality of the dataset and its usefulness to train predictive models of mutational effects on protein stability. In principle, however, a sufficiently general model should not be very sensitive to this problem, as long as it is developed only for prediction on small, soluble, well-folded monomeric proteins with good structures available. However, as discussed in the works commented in the Introduction, in practice, this has not been much the case, with predictions failing importantly on proteins that were not part of the training and validation sets used to train the different methods, only somewhat useful to capture some of the strongly stabilizing and destabilizing mutations.

By design, the S1626 dataset is limited to mutations that induce |∆Tm|< 20 °C, as the authors state that mutations inducing larger Tm changes “are likely to induce important structural modifications”. One could wonder whether such structural perturbations could occur also at lower values of |∆Tm|, say 10 or 15 °C, and we indeed report in subsequent sections examples from the S1626 dataset where structural perturbations are very likely. Conveniently, however, the number of ∆Tm observations in the range from -20 to 20 °C drops smoothly towards both extremes of positive and negative ∆Tm, with very few cases at the ends: only 3 cases between -20 and -19 °C, 6 between -19 and -18 °C, and 3 above + 15 °C. The full distribution of ∆Tm values (Fig. 1C) shows that around 47% are < − 2 °C implying clear destabilization, while only 11.8% are > 2 °C implying clear stabilization, leaving around 41% of nearly neutral mutations (and nearly 25% of the total are within ± 1 °C).

Each entry in the dataset also reports the techniques used to measure Tm. This is dominated by circular dichroism spectroscopy (CD, 46%) followed by differential scanning calorimetry (DSC, 29%) and fluorescence (FL, 18%), with the rest measured through enzymatic activities, absorbance, and other methods (Fig. 1E). The dominance of CD-based measurements is linked to the dominance of lysozyme in the dataset, as all the entries for PDBs 1L63 and 2LZM used this technique. A possible caveat with this variety of techniques is that they are sensitive to different features affected during unfolding; for example, far-UV CD is essentially exclusively sensitive to secondary structure, but fluorescence is rather sensitive to tertiary packing, whereas enzymatic activity can be very sensitive to very local effects such as dynamics around the active site without any effects on folding [24, 25]. We would, however, expect the experimental technique to impact on the raw Tm values but not much on ∆Tm from the wild type, if both wild type and mutant Tm are measured using the same technique as is the case of the entries in this dataset. The distributions of ∆Tm values obtained by CD, DSC, and fluorescence do indeed look similar (Fig. 1F), but anyway, the issue should probably be considered more carefully in future studies and data compilation efforts.

## Learning from the Dataset

**Fig. 2:**
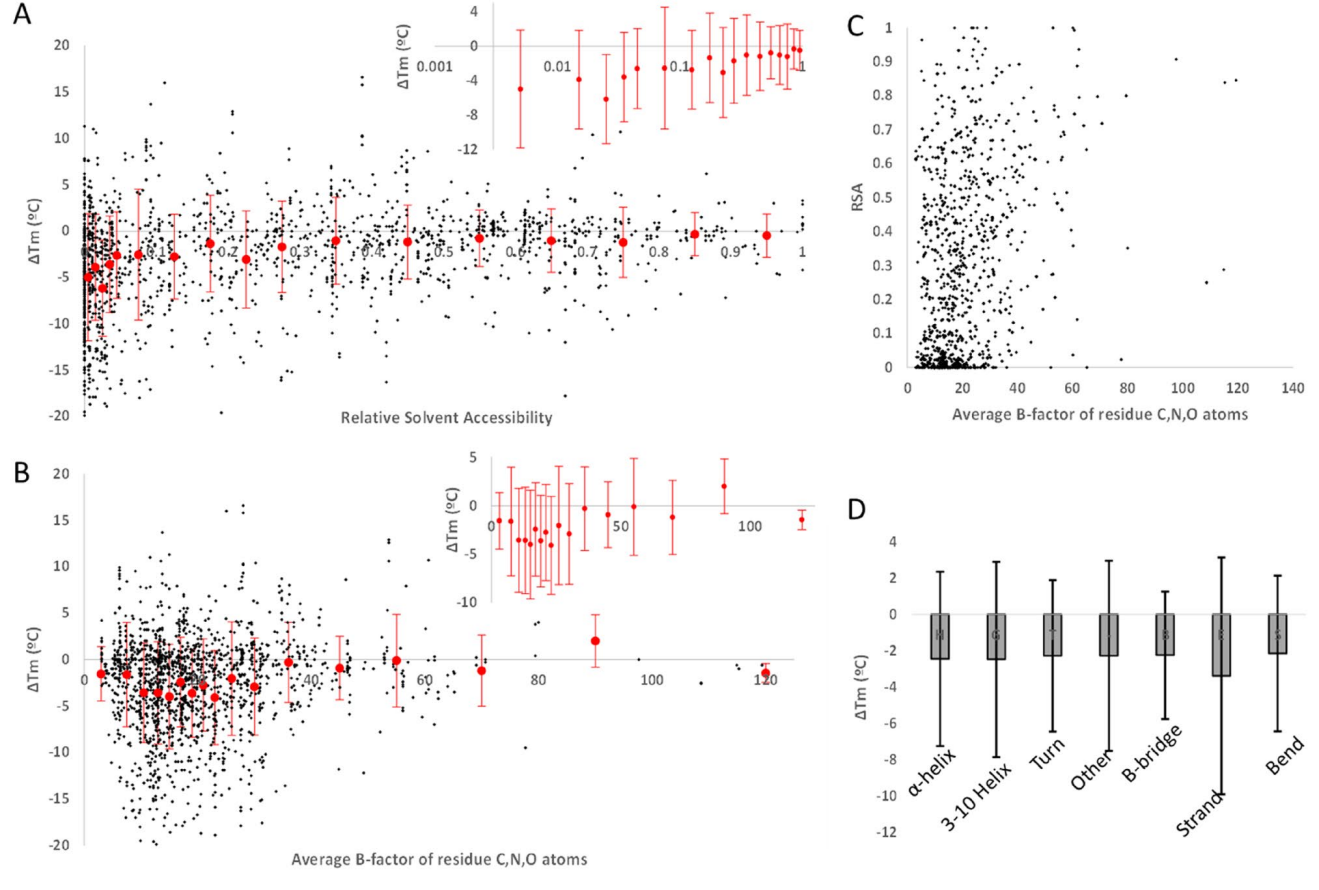
Dependence of ∆Tm entries of the S1626 dataset on protein structural parameters. (**A**) ∆Tm plotted against the RSA of each residue (raw ∆Tm and average ± standard deviation in bins built with similar numbers of entries, the latter shown more clearly in the inset with a logarithmic scale on RSA). (**B**) ∆Tm plotted against the average B-factor of C, N, and O atoms of each residue (raw ∆Tm and average ± standard deviation in bins built with similar numbers of entries, the latter shown more clearly in the inset). (**C**) The Pearson correlation coefficient between RSA and average B-factor is r = 0.3. (**D**) ∆Tm average ± standard deviation for each type of secondary structure identified by DSSP

Analysis of their dataset allowed Pucci et al. to draw some interesting conclusions. First, that most mutations are destabilizing, which are already well documented in literature but are quite quantitatively defined by their data as detailed above. Second, that the fraction of destabilizing mutations seems higher for thermostable proteins compared to mesostable proteins, which is reasonable because thermostable proteins have likely naturally optimized their sequences. In fact, for very stable proteins of the dataset (Tm > 85 °C), the ∆Tm values are all < 5 °C (Fig. 1D). Another finding by Pucci et al., also known but clearly quantified by them, is that mutations at buried residues are in average more destabilizing than mutations at exposed sites, with average ∆Tm of  −  4.3 °C for buried residues (relative solvent accessibility (RSA) < 0.15) and − 1.1 °C for solvent-accessible residues (RSA > 0.5) [11]. A very interesting point here is that the distribution of ∆Tm values is very sharp for exposed residues, most being just neutral to only slightly destabilizing, but quite broad for buried residues as a good fraction of the mutations are very destabilizing and some actually stabilizing. Their paper shows that the distribution width at half of maximum is ~ 4 °C for very exposed residues but ~ 10 °C for buried residues (which represent, respectively, 25% and 45% of the dataset, appropriate to focus the development of predictive models on the buried residues, typically more difficult). That trend is even clearer in Fig. 2A below, which shows increasingly larger stander deviation and lower average |∆Tm| for increasingly buried residues.

McGuinness et al. also reported that, in S1626, residues in beta conformation are more sensitive to mutation compared to other secondary structures, having a more negative average ∆Tm, and that ∆Tm values for residues in coils display a large number of outliers at both positive and negative extremes [17]. A closer analysis based on the detailed secondary structures computed by DSSP does not provide clearer insights (Fig. 2D). But exploring the average B-factor of all C, N, and O atoms of the residues as a proxy for structuring (as less structured residues are more flexible hence have higher atomic B-factors), we find that mutations at more rigid sites are in average more destabilizing than mutations at flexible sites (Fig. 2B), possibly because they are harder to accommodate given the restrained mobility (note that B-factor correlates only weakly with RSA, Fig. 2C).

**Fig. 3:**
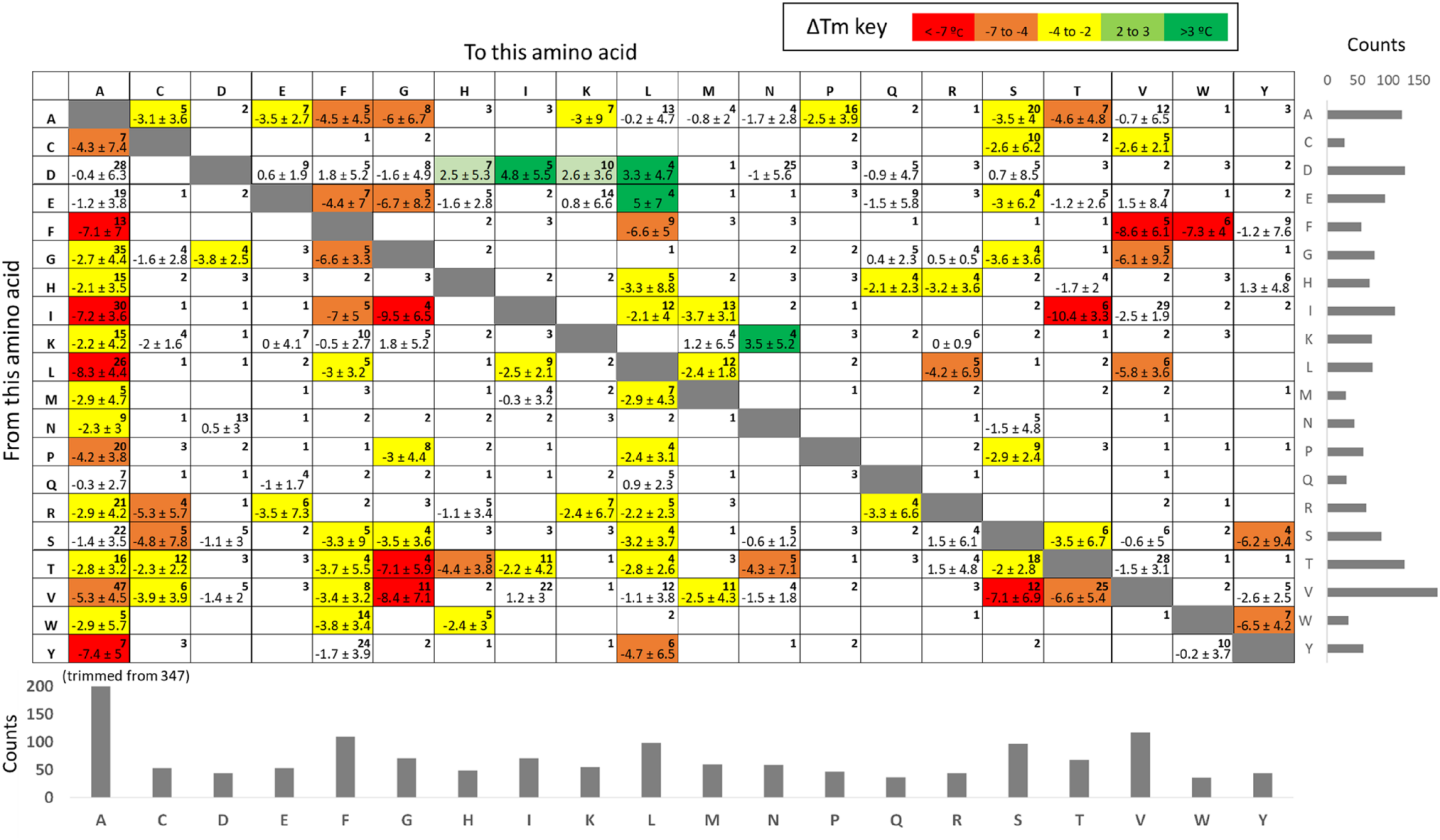
Coverage of all possible wild type- > mutant combinations in the S1626 dataset, and average effects on stability. The matrix shows the average ± standard deviation in ΔTm for amino acid substitutions with more than 3 entries in the dataset, also coded in each cell’s color (from red for destabilizing to green for stabilizing). The number on the top right of each cell counts the number of observations for the corresponding mutation (no number means 0 instances). Below the matrix and on its right, the number of total replacements to and from each amino acid, respectively

It is clear from its authors and from the analysis by McGuiness et al. that the S1626 dataset does not smoothly cover all possible amino acid substitutions. We have analyzed this in some more detail in Fig. 3, further complemented by the subsequent sections. We found that 80% of the 20 × 19 = 380 possible amino acid substitutions are represented by at least one entry in the dataset, and 40% of wild type–mutant pairs’ count with at least 3 entries. For the latter, we present ∆Tm average and standard deviation in Fig. 3. We can easily identify mutations that show up most often destabilizing even if we consider the dispersion of the listed ∆Tm values: mutations from Ile, Leu, and Tyr to Ala all have very negative average ∆Tm, while Phe to Ala also has very negative average ∆Tm although with a standard deviation as large as the average due to one entry with ∆Tm = 9.5 °C (Phe7Ala in PDB 451C, discussed later). Other clear destabilizing effects even considering data dispersion are for example those from Gly to Phe, from Ile, Thr and Val to Gly, from Ile to Thr, from Val to Ser, and from Phe to Val and to Trp. Likewise, we can identify a few mutations that are listed most often as stabilizing from Asp to Ile and Leu (4 entries neutral and 5 stabilizing), from Glu to Leu (2 neutral and 2 very stabilizing), from Lys to Asn (2 neutral and 2 stabilizing), Asp to Lys (4 neutral, 2 slightly stabilizing and 3 very stabilizing), and Asp to His (with only one case of negative ∆Tm among 2 neutral and 4 quite stabilizing cases). Note that as we discuss later, many of these cases may not represent the real, general trends for these mutations. Last, a few substitutions seem to be quite neutral in average, such as those from Gln to Glu which lists 3 neutral cases and one slightly destabilizing case, although the reverse Glu-to-Gln mutation appears quite less neutral. Also substitutions from Gln to Leu and from Gly to Gln result in mild or no impact, while their reverses count few cases so they are difficult to compare. These last observations and other cases involving Gln pose it as a relative inert amino acid regarding stability effects, either if it is mutated or used for substitution; however, it is one of the least covered residues of the dataset so more data are needed to test this proposition.

## A Simple Neural Network Predicts ∆Tm Similarly to Other More Complex Methods, Providing a Baseline that Helps to Separate Global from Structure-Specific Contributions to (de)Stabilization

**Fig. 4:**
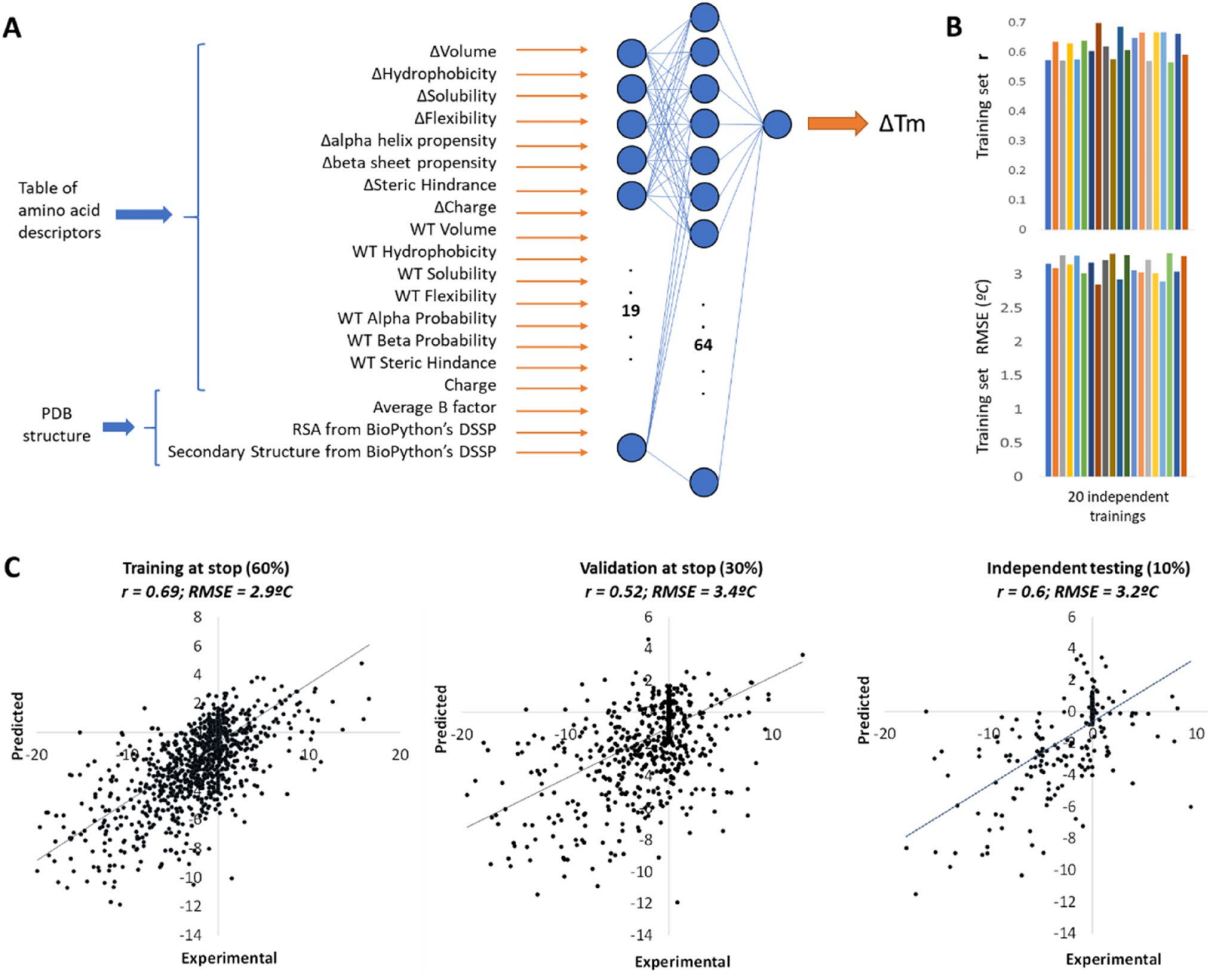
A simple neural network for ΔTm prediction. (**A**) We trained a neural network made of 19 input neurons, 64 neurons in the hidden layer, and 1 output neuron for ΔTm. The inputs are 16 descriptors of the physicochemical changes from wild type to mutant amino acid and their wild-type values taken from the PsychoProt server [40], plus 3 descriptors computed for each residue from the PDB structures (RSA, average B-factor of C, N, and O atoms, and DSSP secondary structure simplified to helical, coil, or sheet). RSA and secondary structures were obtained with DSSP [41]. The inputs were normalized to 0 mean and 1 standard deviation, except for secondary structure that was coded as − 1, 0, and 1 (− 1 for sheet: B, E, S; + 1 for helix helix: H, G, I; 0 for T and others) and charge that was coded as − 1, 0, or 1. The neural network was implemented in TensorFlow 2.5 for Python. (**B**) We trained 20 networks using each time a different random split of the dataset in 60% entries for training, 30% to drive early stop, and 10% for independent testing of the finally selected network. Our whole dataset from which these sets were derived consists in all S1626 entries extended with zeros from 15 randomly sampled mock mutations from wild type to wild type for each protein (which we saw improved results). We used the mean-square error between predicted and experimental ΔTm values as the loss function for training the network. All 20 trained networks yielded similar correlation coefficients (r) and root-mean-square errors (RMSE) between predicted and experimental values. (**C**) We cherry-picked network 11 as our final predictive model. The panel shows correlation plot, correlation coefficient and RMSE for the 3 data subsets, for the final network

Using their dataset, Pucci et al. developed two methods to directly predict ∆Tm upon mutation, which is more directly interpretable and practical than ΔΔGu predicted by most other methods. Their (Tm-)HoTMuSiC models [26] combine information about the amino acid substitution, structural features of the wild-type protein, and the wild-type Tm when available, into statistical potentials that are treated by a neural network trained on S1626 with cross-validation, resulting in root–mean-square error (RMSE) between predicted and experimental ΔTm values of 4.2 °C (or 2.9 °C when outliers are removed). We found that a simple neural network (Fig. 4A) which takes as inputs solely physicochemical descriptors of the amino acid substitutions and the wild-type amino acid, the RSA of the wild-type residue, its local secondary structure, and its flexibility as reflected by the average B-factor of its sidechains, achieves upon training with balanced subsets of S1626 (60% for training, 30% to guide early training stop, and 10% for final evaluation, attempted in 20 different splits that give similar results, Fig. 4B) ∆Tm predictions with RMSE of 3.2 °C and Pearson correlation (r) of 0.6 on the 10% separate testing subset (Fig. 4C). These metrics are very similar to those achieved by HoTMuSiC, both better than other tools for ∆Tm prediction [27–29]. Although these metrics seem to imply reasonably good predictions by our network and other methods, detailed inspection highlights a varying range of deviations. The main issue is that not only our testing RMSE of 3.2 °C on the whole dataset includes 56% cases of very high accuracy with absolute differences < 1 °C between experimental and predicted ∆Tm, but also a substantial fraction of larger deviations: 28% of the entries display differences of up to 4 °C, 10% show differences above 5 °C, and 1% show differences from 10 °C to as much as 16 °C. This bias is barely analyzed in other works, but likely present too, given their similar performance as compared to our network. Also like in other tools, our neural network produces “dampened” predictions where destabilizing mutations are estimated less negative than in the experimental data and stabilizing mutations are predicted less positive, even negative.

**Fig. 5:**
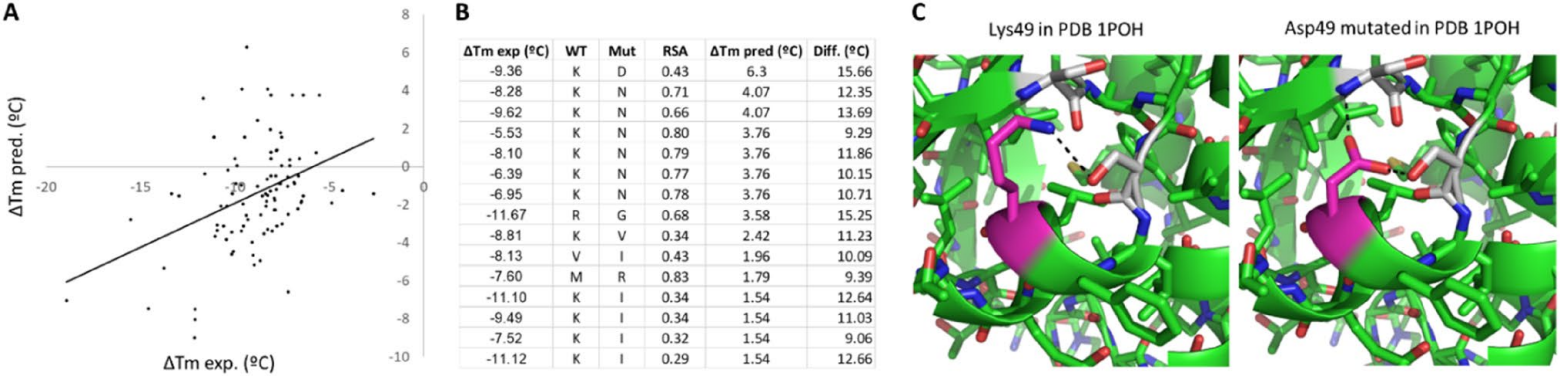
Evaluation of the neural network against the “prospective” data on Guanylate Kinase mutants by McGuiness et al. PLoS One 2018. To predict the Tm changes upon mutations, we needed a 3D model, as there is no structure of this protein available in the PDB, from which we could estimate RSA and secondary structures, while the B-factor was set to 20 for all residues as we lacked data or ways to better estimate it. The 3D model was built through homology modeling of this sequence reconstructed from the information provided in said paper: HHHHHHMALPTPVVICGPSGSGKTTLYNKLLKEFPGVFQLSVSHTTRQPRPGEENGREFHFINRDQFQENIKQGDFLEWAEFSGNLYGTSKKALEEVQANNVIPILDIDTQGVRNVKKASLEAVYIFIKPPSIDVLEERLRSRKTETEEALQKRLSAARNELEYGLKPGNFQHIITNDDLDVAYEKLKGILIKSQMPLAMA. (**A**) Experimental ΔTm against values predicted by our neural network (compare with Fig. 6D of McGuiness et al. 2018). (**B**) Table with all mutations predicted by our network to have ΔTm > 1 °C, showing also RSA, experimental ΔTm, and difference between predicted and experimental ΔTm. (**C**) Lys49 in PDB 1POH (left) and model of its mutation to Asp (right)

We also evaluated our neural network against the “prospective” data on guanylate kinase by McGuiness et al., to find that, just like with the models they evaluated, the network has rather limited predictive power for individual mutations (Fig. 5A). It achieves on this data an RMSE of 8 °C and r of 0.39 between experimental and predicted ΔTm, with differences of at least 5 °C for two thirds of the dataset and a quarter of predictions off by more than 10 °C. Notably, as also found by McGuiness et al. for several stability prediction methods, the network predicts several neutral-to-stabilizing mutations that are actually destabilizing. In the case of our network, these predictions of stabilizing mutations entail the largest differences with the experimental values, and interestingly, they are dominated by mutations from Lys (Fig. 5B). Mutations from this residue are not well represented in the S1626 dataset (except for those to Ala and Phe, see Fig. 3), which probably hampers proper learning by the network. Moreover, mutations from Arg, which could have helped the network to learn by similarity, are also scarce in the dataset. Further complicating the training, the few instances of mutations from Lys to amino acids like Asp and Asn include cases of stabilization which, if arising from specific structural effects, would confuse learning of the actual general trends. And in fact, the strongest deviation in the guanylate kinase predictions is for a mutation from Lys to Asp predicted to be stabilizing by 6.3 °C but actually destabilizing by 9.36 °C (first row in Fig. 5B). The S1626 dataset contains only one case of such mutation, which is quite positive, hence, probably makes the network learn that Lys-to-Asp mutations are in general quite stabilizing. On inspection of this single entry, Lys49Asp in PDB 1POH, we find that Lys49’s sidechain is involved in a hydrogen bond to nearby Ser that requires an unfavorable rotamer (Fig. 5D, left). Replacement of this Lys by Asp results in a stronger hydrogen bond achieved by a favorable rotamer, plus an additional hydrogen bond with a nearby backbone N (Fig. 5D, right). The other stabilizing predictions of the network are dominated by mutations from Lys to Asn, which presents only 4 cases in S1626 (Fig. 3), two of which are nearly neutral and two stabilizing, one actually by 10.7 °C but for which we cannot find any reasonable explanation. Whatever the exact case, more instances of mutations from Lys are required to better model the impact of this apparently important player in tuning stability.

## Interactive Exploration of S1626 through an Online Web App Facilitates Understanding How Structural Details Modulate the Impact of Mutations on Protein Thermal Stability, Helping to Discern General from Structure-Specific Contributions

**Fig. 6:**
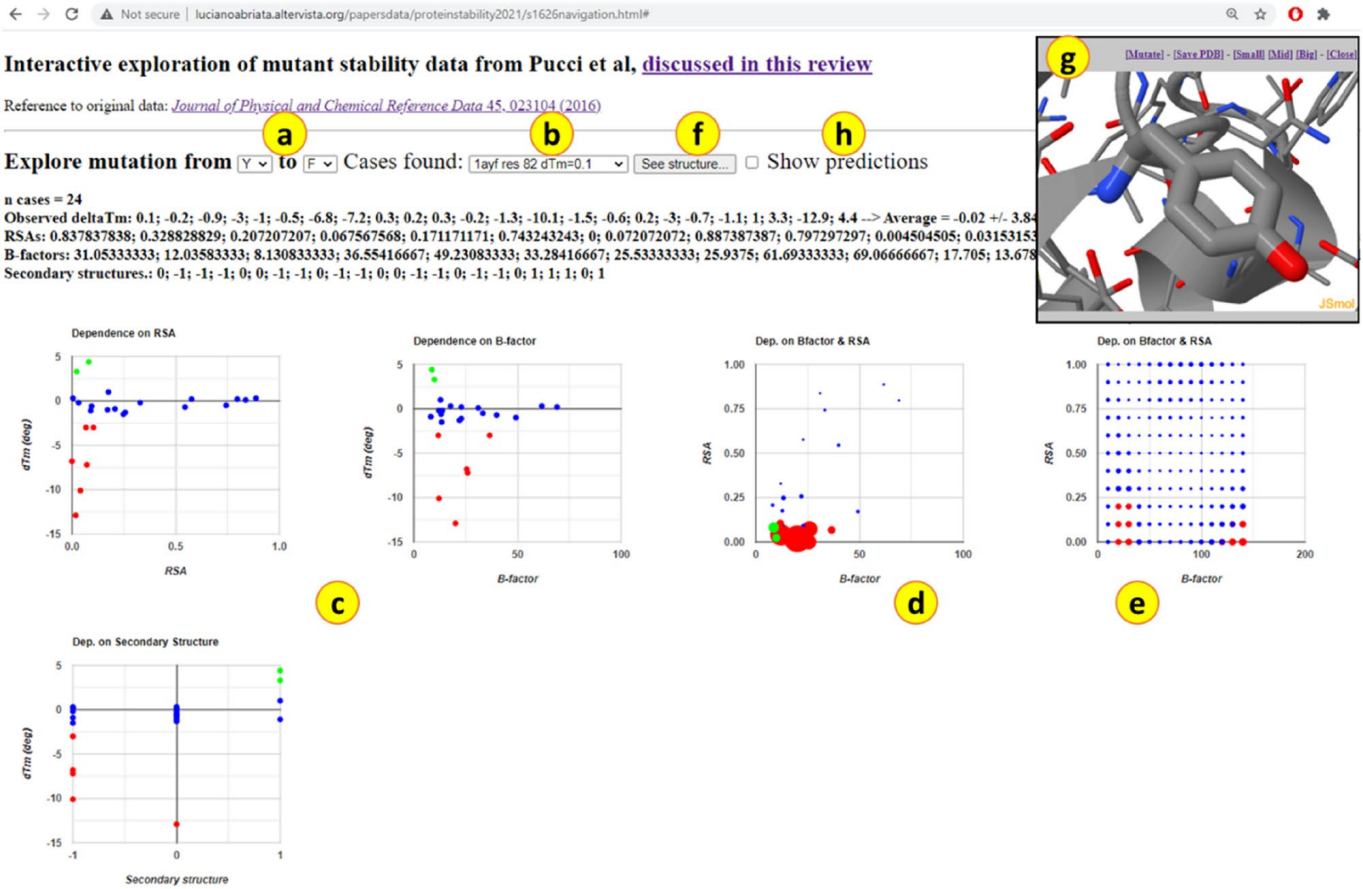
A web app for interactive exploration of the S1626 dataset. When the user choses an amino acid substitution in (**a**) the app presents a list of all entries in (**b**), plots ∆Tm vs. RSA, average B-factor, and secondary structure (**c**), and produces bubble plots of ∆Tm resolved by RSA and B-factor in the entries of the dataset (**d**) or predicted by a simple neural network (**e**). By clicking button (**f**), the app displays in 3D the PDB structure of the currently selected entry, zoomed into the corresponding residue (**g**) which the user can mutate directly on-site. Last, the checkbox in (**h**) shows predictions when active. The web app is available at http://lucianoabriata.altervista.org/papersdata/proteinstability2021/s1626navigation.html

To explore the entries of the S1626 dataset in exquisite detail, we built a web app (Fig. 6) where the entries for each of the 20 × 19 substitutions can be inspected separately, resolved against the structural parameters used above to train the neural network and enhanced with 3D views. Based on web programming [30], this web app is accessible at http://lucianoabriata.altervista.org/papersdata/proteinstability2021/s1626navigation.html on any device. For each possible mutation from one amino acid to another, the app displays plots that resolve ∆Tm against three features of the reference residue as in the structure: RSA as a cue for solvent exposure, average B-factor of its atoms as a proxy for flexibility, and secondary structures simplified from DSSP assignments to either alpha, beta or coil at + 1, − 1 and 0, respectively.

The web app presents data in four plots: one resolving ∆Tm against each of RSA, B-factor, and secondary structure individually, and a bubble plot that displays RSA vs. average B-factor for each entry coding the sign of ∆Tm by color (red for ∆Tm < − 2 °C, green for ∆Tm > 2 °C, and blue for |∆Tm|< 2 °C, i.e., nearly neutral mutations) and its magnitude by the size of the data point. In all plots, hovering over the data points (with a mouse in computers only) displays additional data. Thanks to a built-in JSmol [31] library, users can launch 3D visualizations that automatically focus on the relevant residue, and right there model the mutation and download the mutated file.

**Fig. 7:**
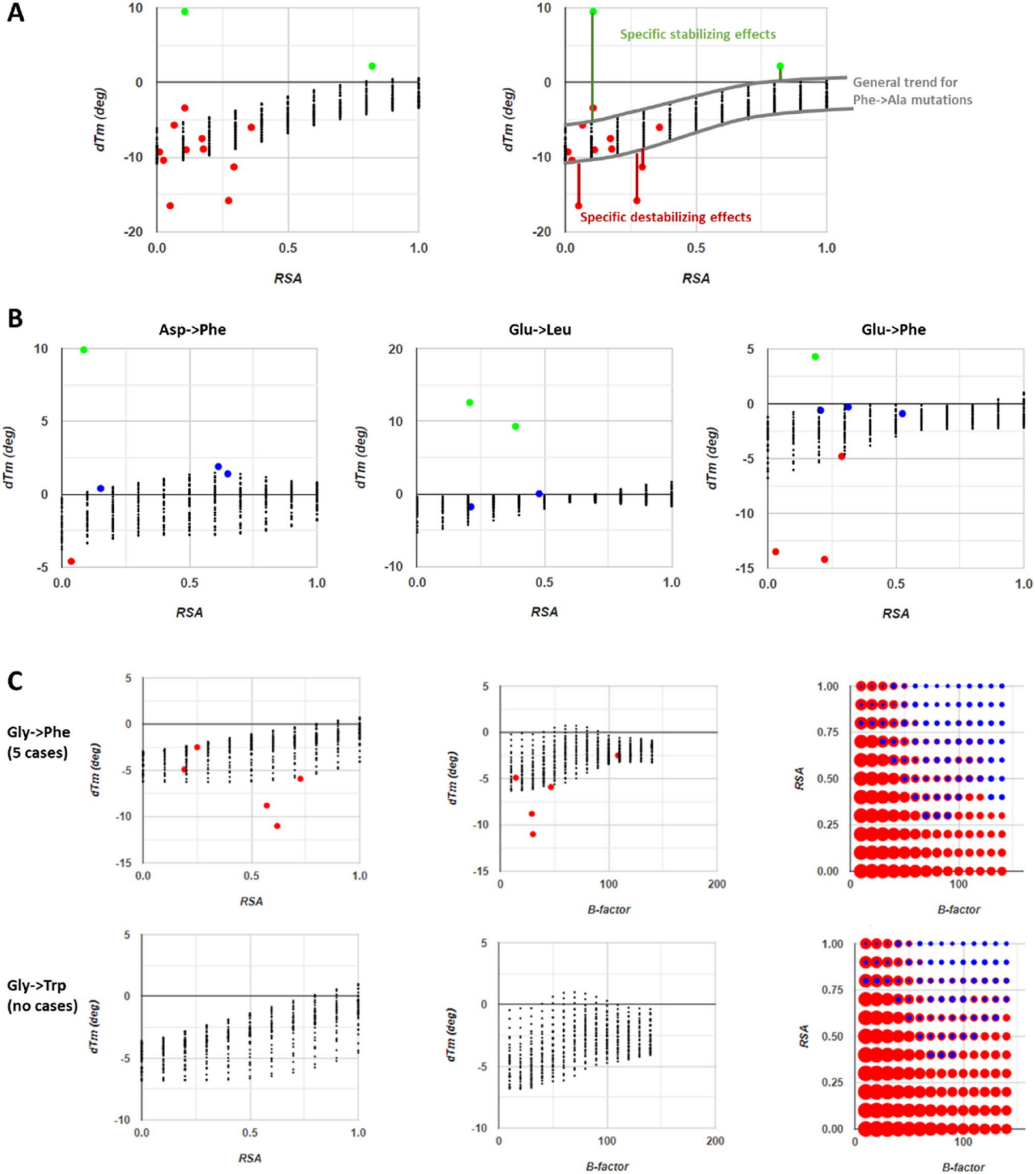
Data analysis and extrapolations guided by the neural network. After running the network on all possible combinations of wild type and mutated amino acids, for all 3 kinds of secondary structures and spanning a wide range of RSA and B-factors, we incorporated the 175,560 predictions into the web app. To view these predictions the user needs to enable “Show predictions” (checkbox h in Fig. 4). (**A**) Left: plot of ΔTm vs. RSA for Phe-to-Ala mutations, showing experimental cases from S1626 in colors as in the previous figures and the network predictions as black dots. Right: the same plot with guides approximating the general dependency of ΔTm on RSA modeled by the network, with strong stabilizing and destabilizing contributions from peculiar structural details. (**B**) Experimental observations and network model of ΔTm against RSA for mutations from Asp to Phe, Glu to Leu and Glu to Phe. (**C**) Dependency of ΔTm on RSA learned by the network for mutations from Gly to Phe, and how this helps it to predict ΔTm for similar mutations that lack any data in the training set, here those from Gly to Trp. For all panels: in the plots of ΔTm vs RSA and B-factor (and secondary structure, not shown in the figure), the network predictions appear as black dots. Meanwhile, RSA vs. B-factor bubble plots encode ΔTm using red for destabilizing, blue for neutral (|ΔTm|< 2 °C) and green for stabilizing mutations, being the bubble diameter proportional to |ΔTm|. When multiple colors are seen this is because of a different output category by different secondary structures of same RSA and B-factor

**Fig. 8:**
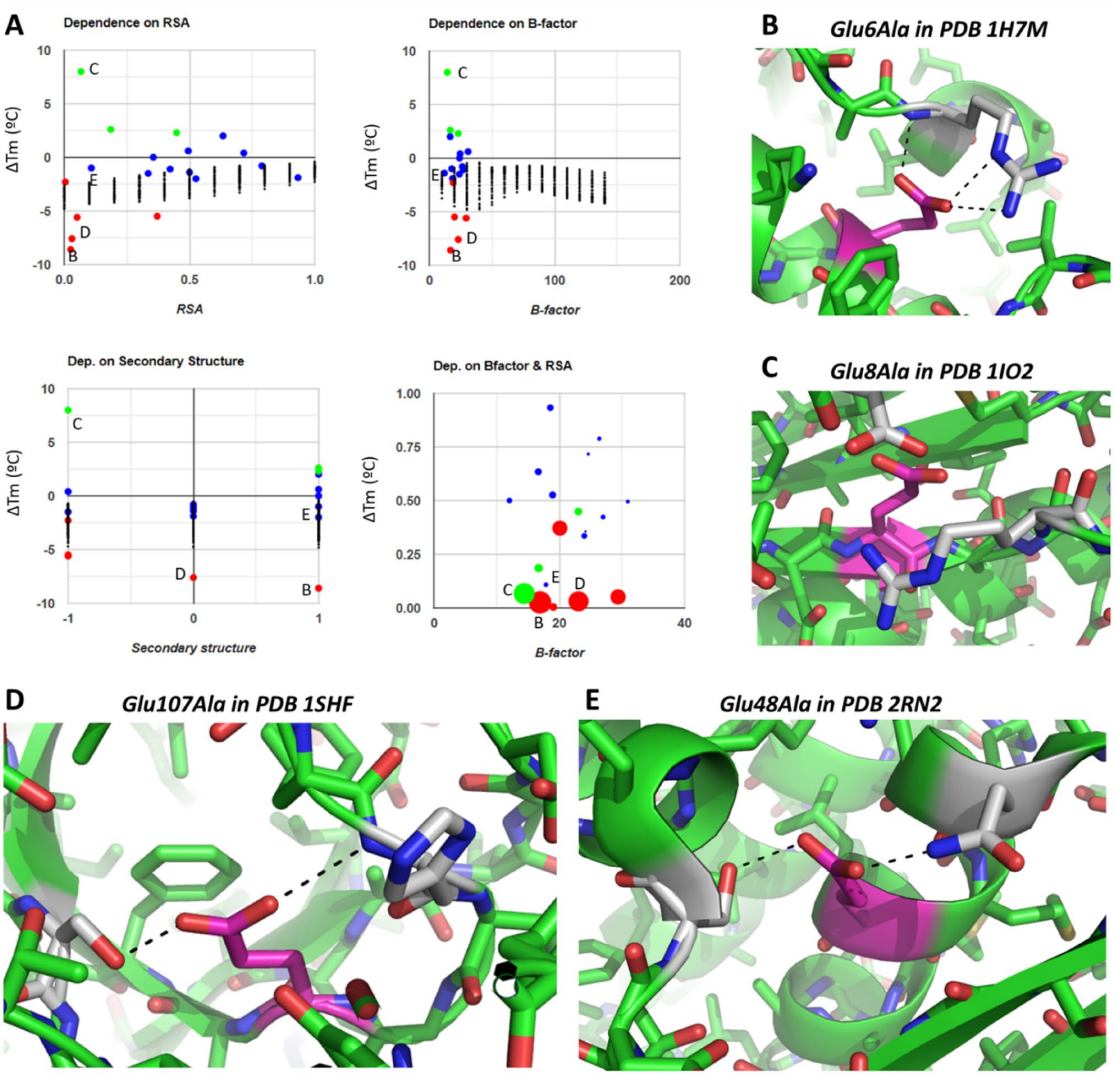
Exploring substitutions from Glu to Ala. In all cases, the residue whose mutation is being discussed is colored with magenta carbons and other relevant residues with gray carbons; all other carbons are green, oxygen atoms red, and nitrogen atoms blue. (**A**) Plots presenting all observations of Glu- > Ala mutations, where letters **B**, **C**, **D,** and **E** relate each data point to the corresponding panel. Red, blue and green colors highlight destabilizing, nearly neutral, and stabilizing entries, respectively, while black dots are the neural network predictions. (**B**) A case of a destabilizing mutation caused by rupture of a salt bridge. (**C**) A stabilizing mutation caused by removal of a charge–charge repulsion. (**D**) A case of destabilization by removal of two hydrogen bonds that connect two parts of the protein. (**E**) A hard-to-explain case, as it resembles (**D**) but in documented as having no strong effect on stability

**Fig. 9:**
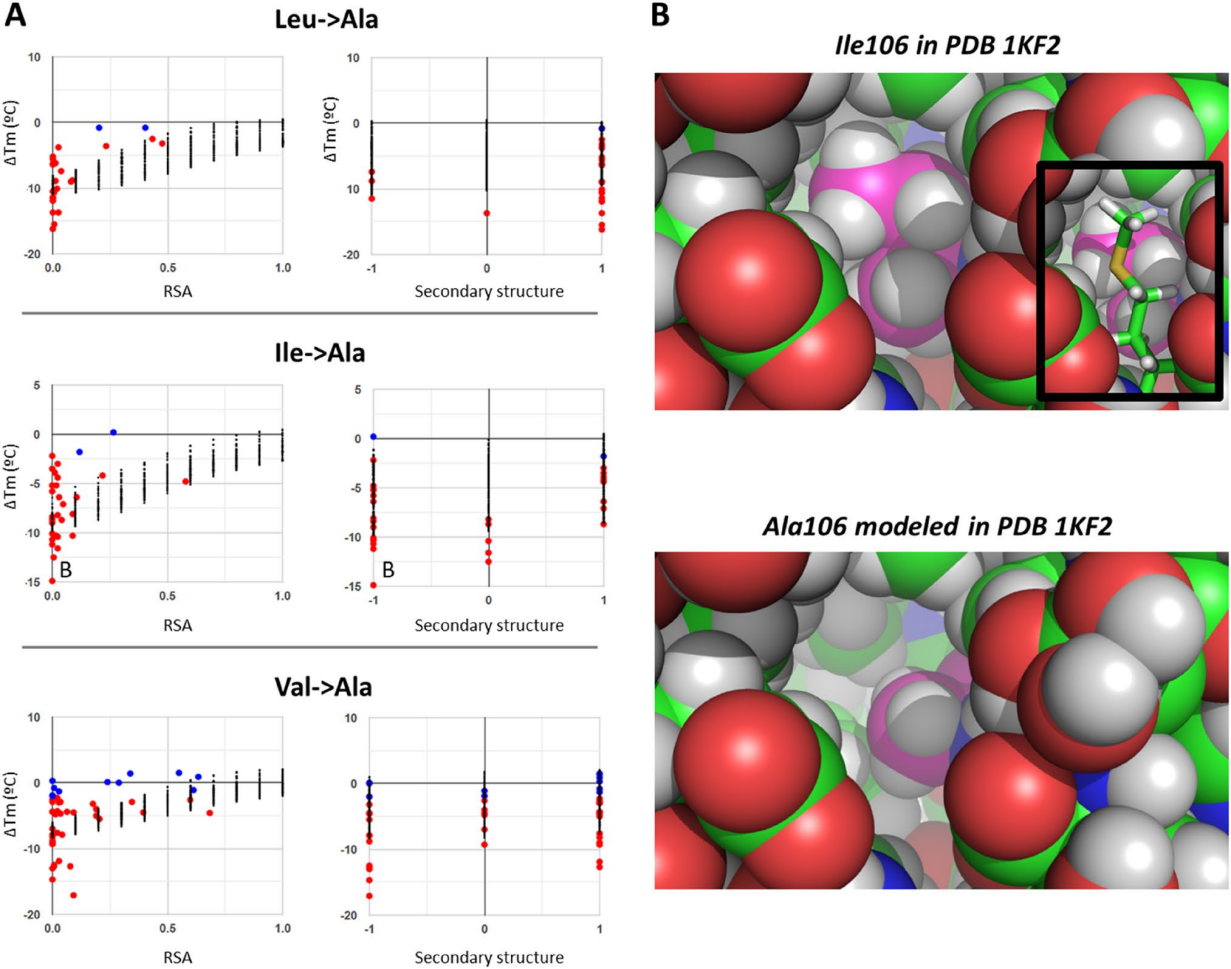
Exploring substitutions from Ile, Leu, or Val to Ala. Atom colors as in Fig. 8, plus hydrogens modeled in white. (**A**) Plots presenting all ∆Tm observations for these three substitutions, resolved against RSA and secondary structure. (**B**) The spacefill models of wild-type Ile106 and the Ala106 mutant of PDB 1KF2 show clearly how Ile fits perfectly at the core, whereas an Ala would leave a void volume that likely causes the observed destabilization. In the models, hydrogens were added to better represent volumes. The inset in the top shows how a methionine, removed for better visualization, encloses Ile106

**Fig. 10:**
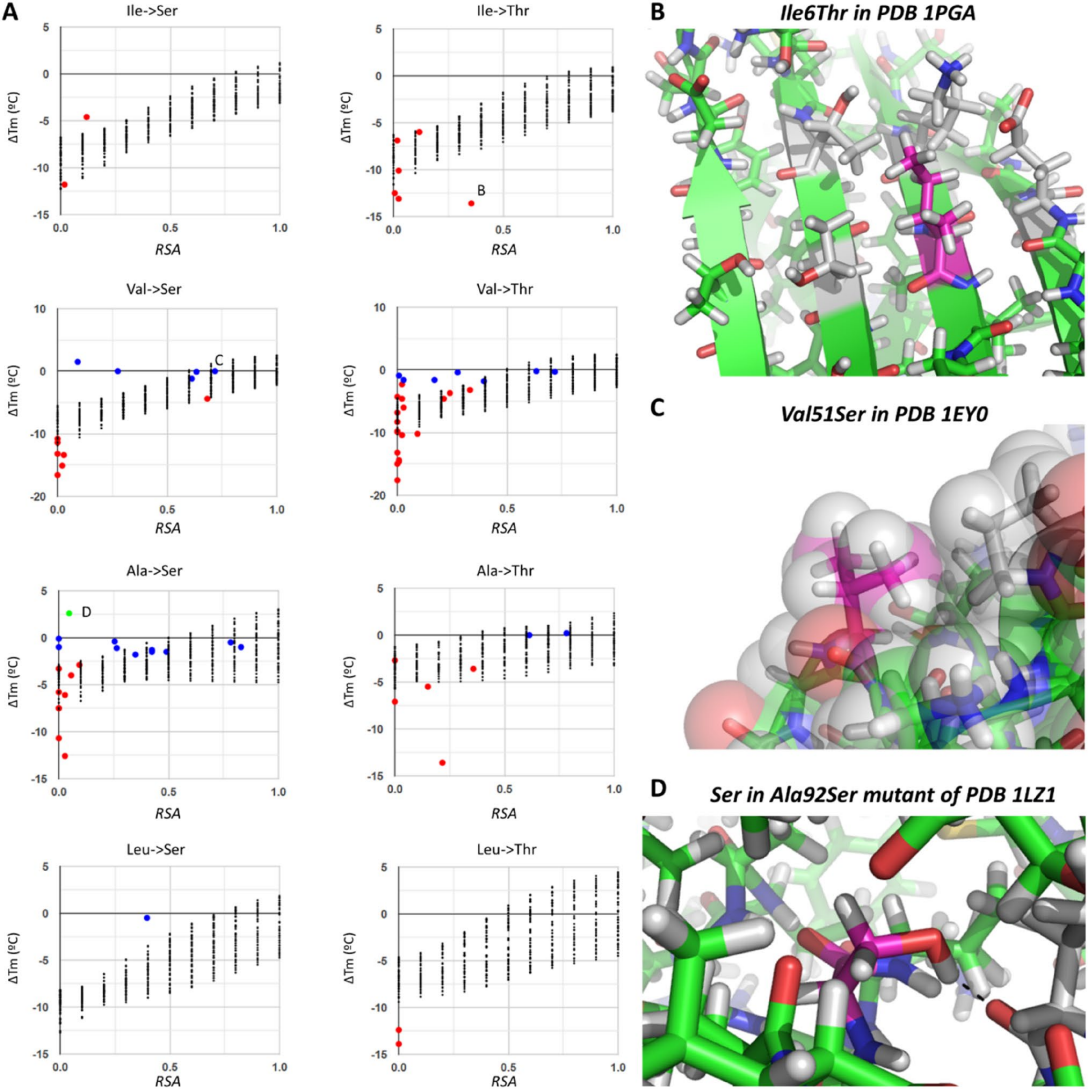
Exploring substitutions from Ala, Val, Ile, or Leu to Ser or Thr. In all cases, the residue in which mutation is being discussed is colored with magenta carbons and other relevant residues with gray carbons; all other carbons are green, hydrogens (added) are white, oxygens red, and nitrogens blue. (**A**) Plots presenting all observations of these substitutions, where letters **B**, **C,** and **D** relate each data point to the corresponding panel. (**B**) The hard-to-explain case of a surface hydrophobic patch, where mutation of the central Ile to Thr results in strong destabilization. (**C**) Another small hydrophobic surface patch formed between a Val and a Pro, but where mutation of the Val to Ser is nearly neutral. (**D**) Ala92 mutated to Ser in PDB 1LZ1 shows how a hydrogen bond is gained at no expense, introducing some stability

**Fig. 11:**
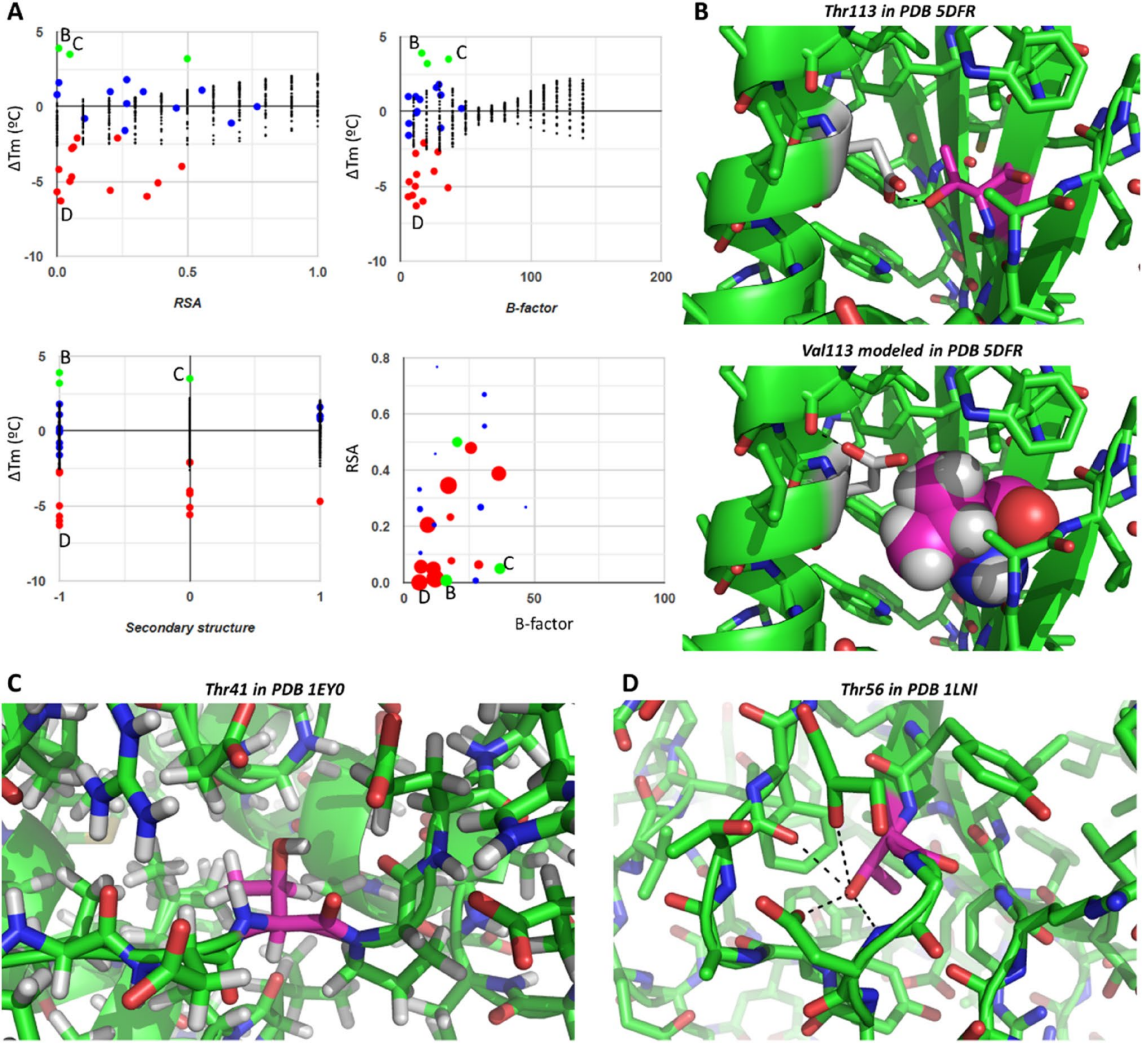
Exploring substitutions from Thr to Val. Atom colors as in other figures. (**A**) Plots presenting all observations of Thr-to-Val substitutions, where letters **B**, **C,** and **D** relate each data point to the corresponding panel. (**B**) Thr113 in PDB ID 5DFR establishes a hydrogen bond with a nearby Asp (top); this interaction is lost when the former is mutated to Val, but the Asp could gain an alternate hydrogen bond if protonated at the same time as the Val methyls pack against the surrounding hydrophobic residues (bottom). (**C**) Thr41 in the quite hydrophobic interior of PDB 1EY0, resulting in stabilization upon substitution of its OH group by a methyl. (**D**) Thr56 in PDB 1LNI is involved in a large network of hydrogen bonds, so its disruption is very destabilizing

**Fig. 12:**
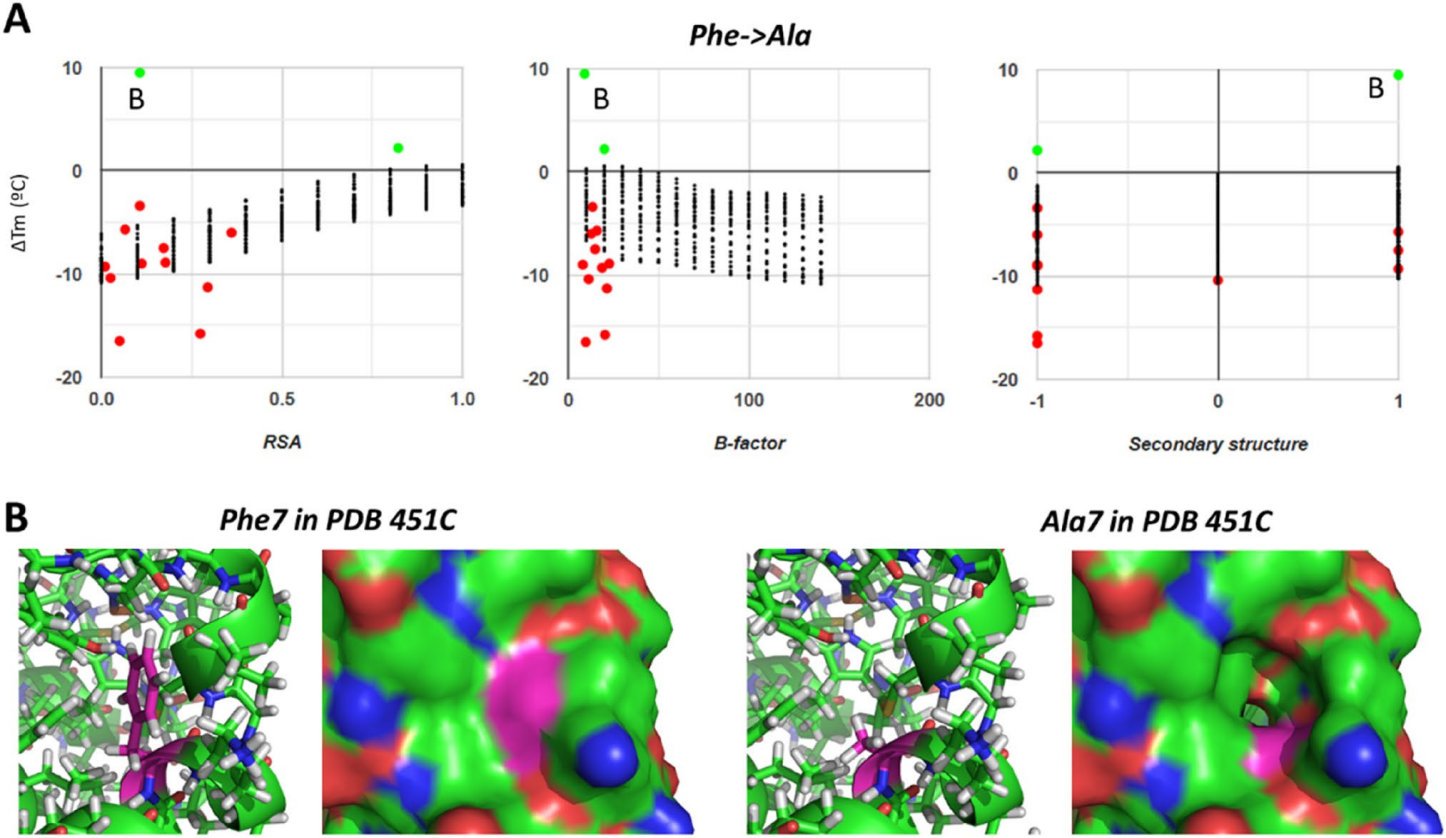
A peculiar case of stabilizing mutation from Phe to Ala, despite a globally destabilizing effect. Atom colors as in other figures. (**A**) Plots resolving ΔTm vs. RSA, average B-factor and secondary structure for all observations of Phe-to-Ala substitutions, where B indicates the case discussed in panel B. (**B**) Stick model and surface representation of PDB 451C centered on Phe7 in the wild-type form or around Ala7 in the mutated form

**Fig. 13:**
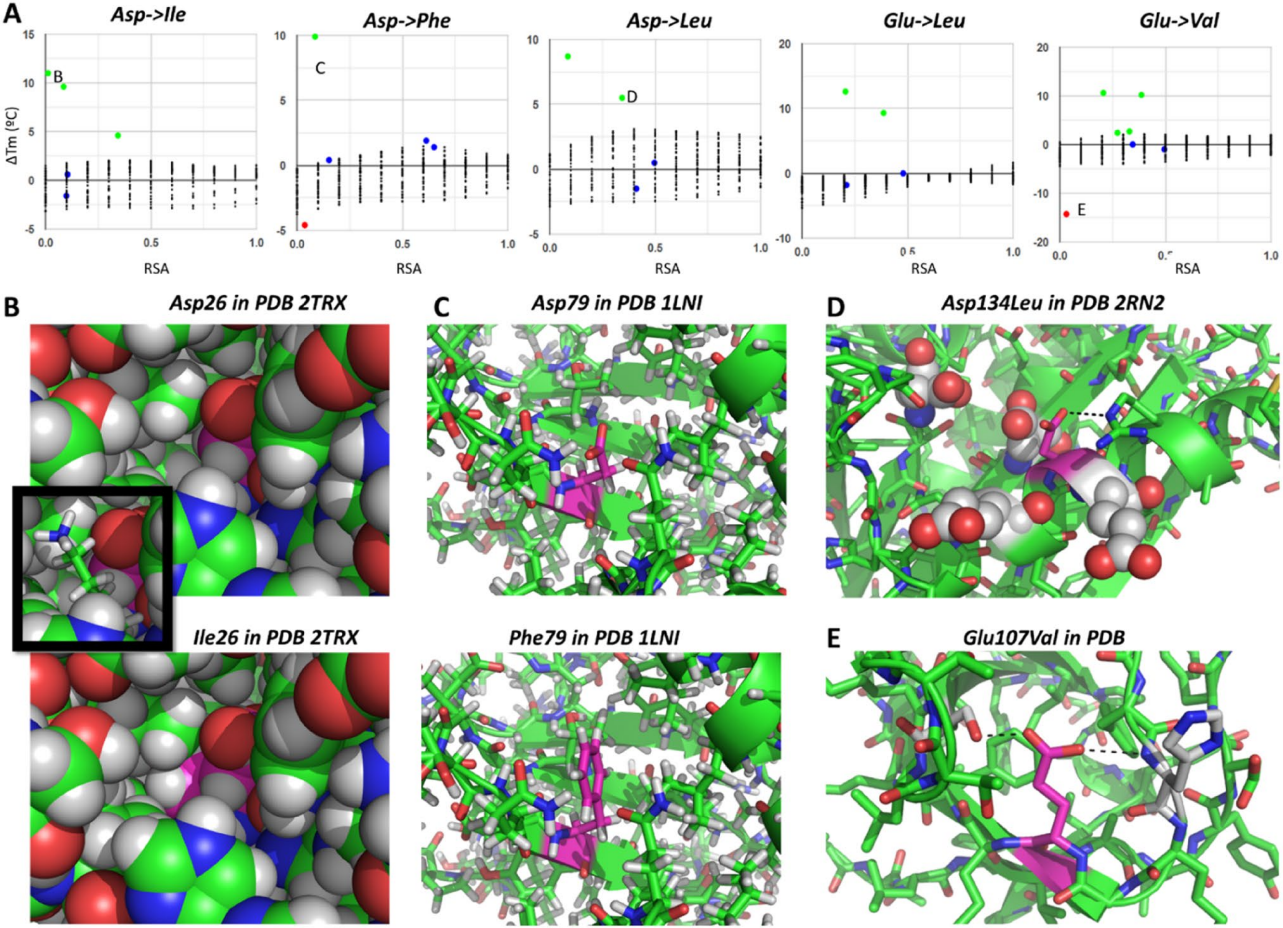
Mutations from Asp and Glu to large hydrophobic amino acids: many unexpectedly stabilizing cases. Atom colors as in other figures. (**A**) Plots resolving ΔTm vs. RSA for the five cases explored, where **B**, **C**, **D**, and **E** point to panels showing the indicated examples. (**B**) Spacefill model of PDB 2TRX centered on Asp26 (top) and its mutation to Ile (bottom); the inset shows a lysine that closes the internal void cavity. (**C**) Sticks models centered on Asp79 of PDB 1LNI (top) and the mutated Phe (bottom). (**D**) Asp134 of PDB 2RN2 surrounded by four other negatively charged residues shown as spheres. (**E**) The hydrogen bonds around Glu107 of PDB 1SHF

At a prediction accuracy similar to that of Tm-HotMusic, an advantage of our network is that it uses simple inputs and runs extremely fast. Thanks to this, we could sample and tabulate all 3 × 11 × 14 = 462 possible combinations of secondary structure (+ 1 for helical structures, 0 for unstructured, -1 for beta-like structures), RSA (from 0 to 1 every 0.1 units), and average B-factor (from 10 to 140 every 10 units) for each of the 20 × 19 = 380 possible amino acid substitutions. We integrated these 380 × 462 = 175,560 predictions into the web app so that users can visualize them overlaid (as black dots) onto the raw entries for each amino acid substitution in the S1626 dataset resolved against RSA, average B-factor, or secondary structure (plots around c in Fig. 6) and also as a bubble map of predictions resolved against both RSA and average B-factor where the sign and absolute value of the predicted ∆Tm are encoded by bubble color and size (indicated with e in Fig. 6). These plots show a recurrent pattern: the network seems to capture a rather general effect caused by each mutation, dependent mostly on RSA that acts as a baseline from which large positive and negative deviations occur. For example, the case of Phe-to-Ala mutations is described by the network mainly as a smooth dependence on RSA such that more buried sites are more sensitive to destabilization by the mutation (Fig. 7A), but it is clear that on top of this general trend, there are other effects that might be quite strong, as exemplified with Phe7Ala in PDB 451C later on. Together with the many structure-specific explanations required to rationalize cases of strongly stabilizing and destabilizing mutations illustrated in Figs. 8, 9, 10, 11, 12, 13 (and others that the reader can inspect in the online web app), the network’s predictions suggest that each individual mutation can be ascribed a general, or baseline, destabilizing-to-neutral contribution natural to the type of amino acid substitution, plus a specific contribution arising from the particular structure around the mutated residue very specific to each case, in which effect can range from very stabilizing to very destabilizing.

As the neural network captures global rather than specific effects (a feature probably shared with other methods, given the similar accuracy), it can then reasonably interpolate and extrapolate the effects of mutations that are underrepresented or even not represented at all in the dataset. For example, mutations from Asp or Glu to the big hydrophobic Phe, Leu, Val, or Ile are dominated in the dataset by neutral and stabilizing examples, although one would expect such mutations to be rather destabilizing. As we discuss later, unfortunately these entries come from very similar substitutions in only a few proteins and represent mainly specific effects. Despite the dominance of positive values for the mutations, the network does deliver rather destabilizing predictions as expected (Fig. 7B). The network probably learns this from the many other instances of similar physicochemical changes produced by other combinations of mutations that are better represented with destabilizing cases. Of course, the positive cases likely do affect the training, such that the actual general effect of these mutations is somewhat more negative than predicted. One further example of how the network produces reasonable extrapolations is that of Gly-to-Trp substitutions, which have no occurrences in the dataset yet are predicted as expected from basic physical chemistry; in fact, the network predicts ΔTm profiles vs. RSA and B-factor similar to those of Gly-to-Phe substitutions (Fig. 7C).

## Detailed Structural Inspections Put Forward Strong Sources of Deviation from Baseline Effects on Stability

In this section and Figs. 8–13, we use the web app to explore the coarse physicochemical rationale for baseline effects of mutations on stability and discuss several specific examples of fine structural details that lead to strong deviations in ∆Tm values. We carry out a very detailed analysis of the potential structural rationales for such deviations, an exercise that is critical to interpret experiments and advance the design of protein mutations as wonderfully exemplified by Castro et al. in their discussion of mutational effects on the stability of human frataxin variants [32].

The first set of examples concerns S1626 mutations from glutamate to alanine, in which ∆Tm values span from roughly − 9 to + 8 °C including a larger fraction of destabilizing and neutral mutations over stabilizing cases (Fig. 8A). The plots suggest that RSA, B-factor, and secondary structure information are not enough to predict ∆Tm, but they do show that for this mutation, the magnitude of the change in Tm (either positive or negative) decreases with RSA, a trend observed for most other amino acid substitutions in the dataset. The two most extreme cases seem to arise from changes in the configuration of buried charges, very important because the electrostatic forces are scaled by a much smaller dielectric constant inside the protein compared to the solvent. One case is Glu6 in PDB 1H7M in which mutation to Ala results in strong destabilization (∆Tm = − 8.6 °C); this residue forms a salt bridge with Arg92 and a hydrogen bond to its backbone, both of which get lost upon mutation (Fig. 8B). On the most stabilizing end, the carboxylate of Glu8 in PDB 1IO2 is very close to that of Glu84, an intrinsically destabilizing arrangement that is lost when the former is mutated to Ala; moreover, such mutation may allow Arg11 to establish a salt bridge with Glu84 contributing further stabilization (Fig. 8C).

For Glu-to-Ala substitutions, ∆Tm is only − 1 to − 2 °C for residues in coil conformation (4 cases) except for one case at − 7.5 °C, Glu107 in 1SHF. This is in a quite buried loop, establishing hydrogen bonds that stabilize closure of the domain; thus, it is not surprising that mutation to Alanine is very destabilizing (Fig. 8D). The other 4 cases of coil glutamates with no large impact on stability upon mutation to alanine are all highly exposed.

But not all cases are easy to explain. For example, Glu48 of 2RN2, quite buried, looks like a stabilizing element as it forms hydrogen bonds with Asn44 one helical turn away and with Ser71 coming farther in sequence. Although this situation is quite similar to the one just discussed, here mutation to Ala is nearly neutral with ∆Tm = − 1 °C with no apparent obvious explanation (Fig. 8E).

The next set of examples (Fig. 9) shows how some similar mutations follow akin patterns of ∆Tm dependence on protein features, here illustrated with substitutions from either Ile, Leu, or Val, all aliphatic hydrophobic, to Ala. All cases entail only neutral or destabilizing effects, and not even one instance of clear stabilization. Destabilizing effects are mild for residues with RSA above 0.15 (all showing ∆Tm > − 5 °C) and range from mild to severe (reaching − 10 to − 18 °C) for residues with RSA < 0.15 (Fig. 9A). Dependences on average B-factor and secondary structure do not add much insight, other than the clear indication that mutations from Ile to Ala in loops are all quite destabilizing with all 5 cases having ∆Tm < − 7.5 °C. The single Leu-to-Ala mutation in coil secondary structure lies also at a low ∆Tm of − 13.7 °C, consistent with the trend for Ile to Ala, while the equivalent coil mutations from Val to Ala are also negative but spanning only from −  1 to −  9 °C. Inspecting structures, it is easy to understand the strong destabilization of many Ile, Leu, or Val mutations to Ala for buried residues, as they are engaged in very hydrophobic clusters where their conformations match perfectly with the surrounding volumes, such that substitutions to Alanine would result in a void space (see example in Fig. 9B for Ile106Ala mutation in PDB 1KF2). Moreover, it is even reasonable that the effects are stronger for Leu and Ile than for Val, because the change in volume from Leu or Ile to Ala is larger than that from Val to Ala. However, the least destabilizing cases are difficult to explain, because they are also engaged in very hydrophobic clusters. For example, Ile15Ala in PDB 1IO2 is only slightly destabilizing with ∆Tm = − 2.2 °C despite looking very similar to the previous case. It is possible that in certain cases, structural rearrangements are accessible that can compensate for the void space that the smaller alanine would create, thus, alleviating the negative impact (in fact Ile15 of PDB 1IO2 is in a loop). This is very hard for programs to predict if they do not consider structural rearrangements, opening a niche for molecular simulations to help.

Replacement of hydrophobic sidechains by small very polar residues also results mostly in destabilization, as exemplified by mutations from Ala, Val, Leu, or Ile to Ser or Thr where again the magnitude of the effect is rather weak for exposed residues but spans all the range from neutral to very destabilizing for buried residues (Fig. 10A). Besides the reduction in volume as in the cases discussed above, substitutions by Ser or Thr bring the additional problem of polar groups being inserted into very hydrophobic environments. In particular, mutations from Leu or Ile to Thr are very disruptive, all 8 cases having ∆Tm < − 6 °C, with even an Ile-to-Thr mutation on a somewhat exposed residue (RSA = 0.36) that reaches ∆Tm = − 13.1 °C. This latter case is very difficult to explain and could probably pose a problem for automated methods, because this Ile forms a solvent-exposed hydrophobic patch leaving no obvious reason for such destabilizing effect of its mutation to Thr (Fig. 10B). Note also the cases where surfaced-exposed hydrophobic residues are mutated to polar amino acids without any gain in stability, as in Val51 of PDB 1EY0 in which mutation to Ser has ∆Tm = 0 °C likely because the Val makes a hydrophobic contact with two methylene groups of Pro56 (Fig. 10C) at the surface.

On the other end of the ∆Tm spectrum, only one of the 73 entries corresponding to these 8 combinations of substitutions is stabilizing, and actually just mildly so an Ala-to-Ser change that increases Tm by 2.6 °C, which could be explained by formation of a hydrogen bond between the introduced OH group and a nearby backbone O while not affecting much the hydrophobic contacts of the alanine’s methyl, now replaced by the serine’s β CH_2_ (Fig. 10D).

Just like there are several cases of Val mutations to Thr, there are also several cases of mutations from Thr to Val (Fig. 11A). Most such cases are neutral to somewhat destabilizing (lowest ∆Tm is − 6 °C), but there are also three somewhat stabilizing entries. The highest ∆Tm is 3.9 °C for the interesting case of the buried Thr113 in PDB 5DFR, which is hydrogen bonded to Asp27. Although mutation to Val disrupts this hydrogen bond, a rotamer change of the Asp is feasible, which would result in a new hydrogen bond to Leu24, while the new methyl group introduced as Val113 packs nicely with the surrounding hydrophobic amino acids and even with the CH2 unit of the rotated Asp27 (Fig. 11B). Another meaningful case of stabilization is that of Thr41 in PDB 1EY0, which is quite buried in a hydrophobic environment, flexible and with no hydrogen bonds satisfied around its OH group (Fig. 11C). Replacement of the OH group by the more hydrophobic and bigger methyl of Val probably stabilizes its position and makes better (hydrophobic) contact with the neighboring residues, resulting in the clear stabilization of 3.5 °C. The opposite case is the replacement of Thr56 in PDB 1LNI, in which OH group is involved in several polar contacts, by the hydrophobic Val resulting in a destabilization of − 6.3 °C (Fig. 11D).

As mentioned when discussing Fig. 3 and described with many examples above, mutations that cause large changes in volume tend to be quite destabilizing. Although this is much expected from basic physical chemistry, there are important outliers of quite stabilizing mutations, as exemplified by one of the Phe-to-Ala substitutions in Fig. 12A. Baseline destabilization in Phe-to-Ala mutations likely arises from the void space that would result upon substitution if the structure cannot rearrange. But mutation of Phe7 to Ala in PDB 451C shows positive ΔTm by 9.5 °C (checked against the original publication [33]). This residue establishes several hydrophobic contacts and is quite buried, with an RSA = 0.11 that makes it better buried than many of the Phe-to-Ala mutations that have destabilizing effects (Fig. 12B). Mutation of this Phe to Ala removes the hydrophobic contacts and would create a quite large cavity at the surface, which one would in principle assign as destabilizing. One way to explain the actually stabilizing effect is that the cavity is sealed through structural rearrangements that end up repacking the hydrophobic residues around Ala7 even better than around Phe7 in the wild-type protein. This would then be an example of mutation that only methods with substantial structural sampling can potentially capture. Importantly, this and similar cases presented in this review argue against Pucci et al.’s premise that substitutions with |ΔTm| in the range from 10 to 20 °C still entail no major structural perturbations.

We conclude this section of examples, which the reader can extend using the web app to all other amino acid substitutions with data, with mutations from Asp and Glu to large hydrophobic amino acids like Val, Leu, Ile, and Phe (Fig. 13). Chemical intuition would suggest that these should be quite destabilizing, but the dataset reports only one such case. This single destabilizing case is mutation of Glu107 to Val in PDB 1SHF (Fig. 13E), a residue that forms two hydrogen bonds that get lost upon mutation, just like in its mutation to Ala in Fig. 8D but here aggravated by the larger volume of the Val sidechain (ΔTm to Ala is − 7.5 °C and to Val is − 14.3 °C). Of the other 24 cases of mutations from Asp and Glu to Val, Leu, Ile, and Phe, 11 are just neutral and 12 are stabilizing, some even reaching quite high ΔTm. Inspection of the more extreme cases shows sources of stabilization that seem quite specific to each structure. In the mutation of Asp26 to Ile in PDB 2TRX, the starting structure has a void space where the extended sidechain of Ile fits perfectly upon modeling, thus, filling in the cavity without the need of structural rearrangements (Fig. 13B). The case of Asp79 to Phe in PDB 1LNI not only is harder to explain but might also arise from better filling of a cavity by the Phe side chain at the expense of no hydrogen bonds around the Asp carboxylate (Fig. 13C). In fact, another mutation of this residue but by Val is even more stabilizing; however, this is easier to explain because the mutation preserves the shape removing a charged group from a hydrophobic environment and placing methyl groups instead. The last example, Asp134 in PDB 2RN2, is engaged in a salt bridge that would get lost upon mutation but could be compensated by hydrogen bonding to a nearby Glu, thus, having no net effect. However, Asp134 itself is surrounded by other four negatively charged groups, so its mere replacement may bring alleviation of the repulsions between all these negative groups, resulting in the observed stabilization that works also with other mutations that remove the negative charge available in the dataset.

## Considerations Regarding the Development of ΔTm Prediction Methods and New Training Datasets: Review of Established Ideas Convoluted with Proposals Based on our Analyses

### Refining the Need for Larger, Better ΔTm Datasets

Papers and reviews reviewed in this work highlighted the need for datasets with better coverage of all possible mutations, to which here we add the need to also better cover the space of structure-dependent features around each mutation. Given that our network performs similar to other methods and programs for ΔTm estimation, it is likely that the observations discussed throughout this work apply to them as well. The following recommendations are hence likely of interest to all developers and users of such methods. As we have shown for several kinds of substitutions, one clear example being those from Gly to Trp in Fig. 7C, even simple prediction methods like our neural network can fill in certain gaps of the dataset with reasonable approximations; however, it is also clear that overall predictions are quite off for many of the 20 × 19 substitutions, especially when effects from rather special structural details dominate the training set, thus, confusing network training as shown for mutations from Lys to Asp among other examples. Clearly, more observations in other proteins are required to better establish the general component of the stability effects for these mutations and to tell if these substitutions are overall as stabilizing as the entries of S1626 suggest or, rather more likely, the specific entries listed are not representative of the general trends. But how much data are enough?

**Fig. 14:**
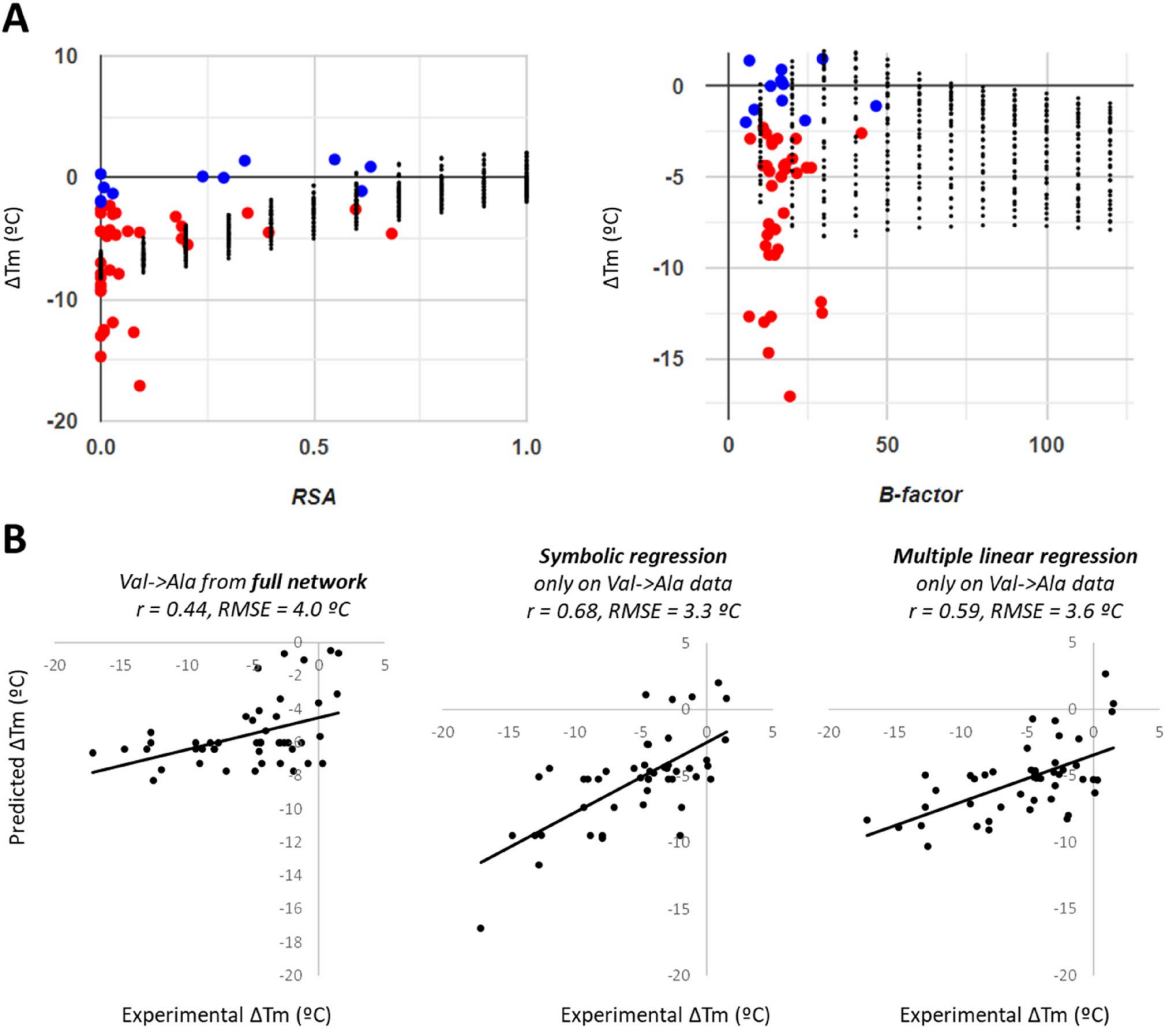
Modeling the 47 observations of Val- > Ala mutations. (**A**) Plots of ΔTm dependence on RSA and average B-factor for mutations from Val to Ala, from the web app. (**B**) correlation plot between experimental ΔTm and ΔTm predictions for all Val-to-Ala mutations of the dataset. Left: from predictions by the neural network of Fig. 12; center: from symbolic regression on RSA, average B-factor, and secondary structure; right: from multiple linear regression on RSA, average B-factor, and secondary structure. Symbolic regression was carried out using 70% of the Val-to-Ala entries for training and 30% for validation. It produced the equation ΔTm (°C) = SS–SS / (8.58 RSA–0.89) + 13.56 RSA–7.35. The linear regression was carried out on all Val-to-Ala cases, resulting in the equation ΔTm (°C) = 12.63 RSA–0.089 B-factor + 1.95 SS–5.81

To estimate how many observations are “enough” in a sufficiently complete dataset, let us consider the case of Val-to-Ala substitutions, which is the best represented mutation with 47 entries well spread in RSA between 0 and 0.7, B-factor between 0 and 50 which is as much as Valine residues get in folded proteins, and the three main secondary structures (Fig. 14A). On this mutation only, the network achieves a RMSE of 4.2 °C, a correlation r = 0.41, and quite dampened predictions (Fig. 13B left). However, symbolic regression [34] on RSA, average B-factor, and secondary structures as independent variables can model the data analytically achieving r = 0.68 and RMSE = 3.3 °C, even having split the 47 entries into 33 (70%) for training and leaving the rest as a check subset to stop model training. In principle, with enough data for all possible mutations, it could be possible to build similar analytical models for the general contributions to stability changes in all of them, with the advantage of being more interpretable than neural networks which rather behave as black boxes. For the example shown for Val-to-Ala mutations, the fitted equation (see caption for Fig. 14B) reveals a strong dependence on RSA followed by some effect of secondary structure and no distinguishable contribution from B-factors. A simpler alternative is to perform a linear regression, which results in slightly worse prediction of the stability changes yet better than that of the full neural network (Fig. 14C, right). In this case, the regression equation also detects RSA as the main contributor variable, with an offset of -5.81 °C, essentially no contribution from B-factors (which reach 5–50 times larger values but has a regression coefficient around 140 times smaller), and some contribution from the secondary structure. This interpretability is similar to that reported by a novel work showing that multilinear regression models on three simple parameters (RSA and differences in volume and hydrophobicity between wild-type and mutated residues) can achieve very simple, human-interpretable predictions of ΔΔGu at accuracy similar to that of other programs [35].

We tested similar symbolic and multiple linear regressions for other substitutions of the dataset, but none resulted in such large improvements relative to the full neural network and only Gly to Ala and Ser to Ala could roughly capture some of the experimental trends, although the effects here are only mildly destabilizing and the equations derived through symbolic regression are quite more complex than that for Val to Ala, suggesting possible overfitting. For comparison to Val-to-Ala mutations, mutations from Gly to Ala and from Ser to Ala count with 35 and 22 observations, respectively. Arguably, substitution from Val, Gly, or Ser to Ala are among the easiest cases, so modeling other substitutions properly may well require even larger numbers of examples, especially to cover the different possible structural details that induce strong effects.

Having larger number of observations would also help to better define those mutations that seem, based on the limited dataset, to never induce strong effects on stability, as apparent from Fig. 3 for some mutations involving Gln although at the moment counting with too few cases for generalization. New datasets like FireProtDB [14], ThermoMutDB [12], and the latest ProTherm [13] are very promising to alleviate the problem of poor mutational coverage, although for our specific problem of predicting ΔTm, they all suffer from incompleteness, as most entries contain only either ΔTm or ΔΔGu data and many entries include multiple simultaneous mutations. It is possible that rather than creating and curating new datasets, the community needs a coordinated effort to properly map, experimentally, all the scarcely covered substitutions on at least a defined set of proteins. Just completing all cells of the substitution matrix of Fig. 3 for small, soluble, globular, two-state folder proteins would be a major effort which would deliver a dataset highly specialized but at least very useful for, these proteins. Such an effort should include multiple situations for each possible substitution, so as to properly capture not only the general effects of each type of mutation, which are somewhat already accounted for by the S1626 dataset but also the effects of different structural subtleties that may be quite strong as we have shown in multiple examples. Based on all cases analyzed here, the list of structural details to consider include minimally internal salt bridges and hydrogen bonds not only formed or lost but also those rearranged upon mutation, changes in sidechain volume that might be less destabilizing than expected or even stabilizing if they are compensated by structural rearrangements, (re)packing of hydrophobic clusters not only at proteins cores but also right underneath protein surfaces and relaxation of densely charged regions.

### Remarks on Method Development

**Fig. 15:**
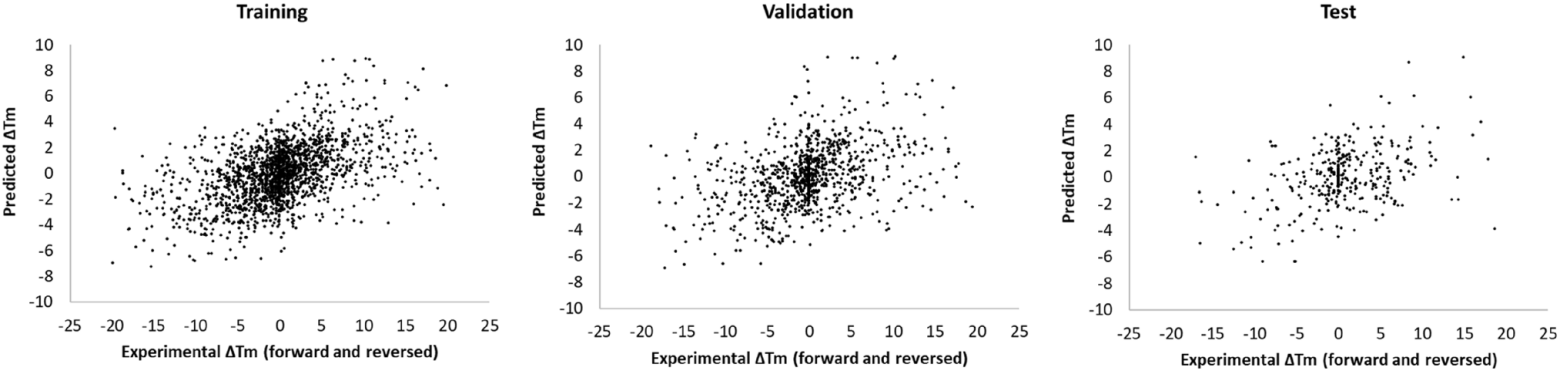
A neural network like that of Fig. 4 but trained on the full S1626 dataset plus all the reversed mutations. From left to right, plots show correlations between predicted and experimental values in the training, validation, and testing sets (respectively, 60%, 30%, and 10% of the whole dataset of forward plus reversed mutations). Correlation coefficients are, respectively, 0.49, 0.39, and 0.42, and RMSEs are 4.6, 5.0, and 4.9 °C

An important aspect which seems obvious but has only recently been addressed, and so far only for ΔΔGu predictions although also applicable to ΔTm predictions, is that predictions should be symmetric when comparing forward to reverse mutations. This means ensuring that ΔΔGu (and also ΔTm) predicted from wild type to mutant and vice versa should be of same magnitude and different sign. As Pucci et al. showed, this is hardly the case for most ΔΔGu predictors, a problem that stems in the bias of training datasets towards destabilizing mutations [16] to which neural network-based systems are especially prone. Work by these authors also showed that certain physical symmetries can be imposed to correct for this problem, although of course at the expense of some prediction accuracy [16, 36]. It remains to be tested if similar approaches can also correct forward-reverse symmetries in predictions of ΔTm. In our hands, training networks just like that presented in Fig. 4 but including all reversed mutations on top of the forward mutations, and assuming the same RSA, average B-factor and secondary structures as in the wild type, results in a correlation coefficient of 0.42 and an RMSE of 4.9 °C in the independent test set (Fig. 15). These numbers are substantially worse than the correlation of 0.6 and RMSE of 3.2 for our network trained only on the forward data but is still good enough to capture global trends, now not only destabilizing ones but also those that are stabilizing. Furthermore, this prediction capacity can probably be improved by modeling the mutations to obtain better input RSA values.

Last, an obvious, important conclusion of our analyses is that methods that treat structure explicitly have better chances of capturing the complex structure-dependent effects of mutations on (de)stabilization. As we have shown through examples, this might be important especially for mutations from and to amino acids of drastically different volumes and, not minor, may be important already for changes that induce |ΔTm| of already 9–10 °C, i.e., quite before the usually accepted limit of 20 °C. Properly modeling structural perturbations induced by mutations are far from trivial yet critical, because as many of our investigations show a simple backbone displacement or rotamer can change whether a hydrogen bond is lost, gained, or swapped. The incorporation of molecular dynamics simulations is enticing, especially as force fields evolve [37], because it would enable structural relaxations that are otherwise very difficult to predict. The downside of such simulations is that they are very costly in terms of computer time. Less detailed but far more efficient methods using normal mode analyses may find some utility in cases where changes in flexibility modulate changes in stability, as exploited in the DynaMut method for predicting the impact of mutations on ΔΔGu [38].

Yet it is important that many caveats will still stand even if we get very detailed, complete datasets and employ complex methods based on simulations; for example, changes in stability originated by changes in oligomerization states (especially important when mutations affect surface hydrophobicity as in certain cases presented) will be very difficult to account for, because not even the most complex simulations can capture this correctly. Homology modeling at very high sequence similarity, i.e., essentially a problem of sidechain rotamer optimization, seems today to work quite well to predict melting temperature change. However, homology modeling already at sequence similarities under 98% already results in quite substantial loss in the quality of predictions [39].

## Footnotes

1 One was lack of maintenance since 2013; however, a new version of ProTherm just came out in 2020 as presented by Nikam et al.

2 We note that Huang et al. did find that some programs are good enough to at least tell strongly stabilizing from strongly destabilizing mutations.
